# Low-Temperature Prediction in Commercial Lithium-Ion Batteries during Dynamic Usage via Enhanced Physics-Informed Neural Networks

**DOI:** 10.1021/acsomega.5c11152

**Published:** 2026-05-13

**Authors:** Eric L. Pereira, Davi M. Soares

**Affiliations:** Department of Electrical and Computer Engineering, 8683 Wichita State University, 1845 Fairmount Street, Wichita, Kansas 67260, United States

## Abstract

Monitoring lithium-ion battery (LIB) temperature in real time is crucial for ensuring system safety, especially across different chemistries, testing, and environmental conditions. In particular, low temperatures, accurate temperature prediction is crucial because it supports early detection of potentially unsafe operating conditions, such as those associated with lithium plating. From such prediction, the battery management system must intervene before any damage to the LIBs occurs, thus improving both safety and performance in cold environments. This work introduces an enhanced physics-informed neural network (PINN) model that leverages thermal laws and entropy effects to accurately predict the surface temperature of commercial LIBs cycled at 1C at different temperature conditions (5 °C, 25 °C, and 45 °C). As an understudied temperature range, focus is given to low temperature cycling. Unlike prior PINN approaches, the proposed model incorporates Joule heating, reversible entropic heat, and radiative losses directly into its loss function. The model also uses adaptive weighting to balance physical constraints with data-driven terms. A charge-consistency term further enhances prediction stability by linking state of charge behavior to thermal dynamics, while enforcing unit consistency in the final loss function. The experimental results from three commercial cell chemistries (LFP, LCO, and NMC) show that the proposed model achieves mean absolute errors as low as 0.07 °C (LFP at 5 °C). Such performance was obtained when the model is trained and evaluated within the same chemistry using only 30% of the dataset. These results fall within the typical uncertainty range of thermocouple measurements (±1 to ±2 °C), demonstrating that the proposed approach is robust and shows close agreement with the measured temperature signals, thereby supporting improved safety and performance in lithium-ion battery systems.

## Graphical abstract

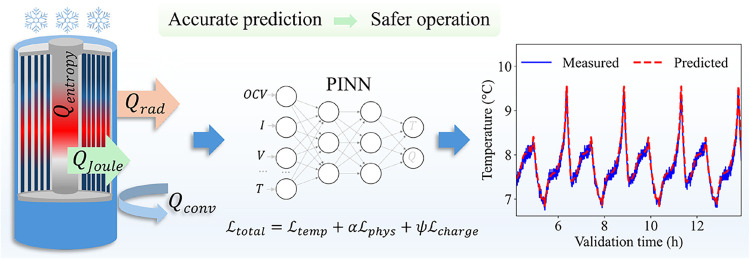

## 1 Introduction

Lithium-ion batteries (LIBs) are a key technology for electric vertical takeoff and landing (eVTOL) aircraft, stationary energy storage systems for artificial intelligence (AI) data centers, and portable electronics.^1–4^ LIBs provide excellent energy density, are small and light, and can store energy from solar and wind sources. In addition, LIBs market is projected to grow to over USD 187 billion by 2032, driven by demand across the full supply chain.^5,6^

As LIBs become relevant for modern energy and transportation systems, ensuring that they are at a safe state is paramount. Among the various factors that influence LIBs operating limits, the thermal behavior is also a critical determinant for performance, safety, and longevity assessment.^7^ The performance and lifetime of LIBs are strongly influenced by operating temperatures.^8^ Both very low and high temperatures may accelerate degradation modes.^9,10^ At low temperatures, LIBs suffer from sluggish electrochemical kinetics and increasing internal resistance.^11,12^ These conditions increase capacity loss, accelerated degradation, and the risk of lithium plating, which seriously restrict the operating conditions of LIBs in cold environments.^11^ In these conditions, the polarization increases and the discharge voltage falls, causing major energy loss. Lithium-ion intercalation and deintercalation is slowed down, which lowers Coulombic efficiency and makes charging and discharging difficult, shortening the useful life of the battery.^11,13–15^ Charging at low temperatures may cause lithium plating and dendrite growth on the anode, that can penetrate the separator and cause short-circuit, resulting in rapid heat generation or, even lead to thermal runaway and explosion.^16–18^

Overall, the temperature evolution during cycling provides an indirect indicator of these adverse processes, since abnormal thermal responses may signal inefficient charge transfer or side reactions—such as dendrite formation.^19^ Therefore, a reliable model that captures temperature behavior under cold conditions is urgently needed to support safe operation and informed control decisions. For instance, this information allows control strategies in a battery management system (BMS) to adjust charging profiles (derating) and prevent permanent damage to cells. However, the mathematical modeling of lithium-ion batteries plays an important role in estimating their internal states and supporting thermal management. Since battery performance is strongly affected by temperature, nonlinear effects often arise. While most existing models provide reliable predictions under room or elevated temperatures, their accuracy declines noticeably under low-temperature conditions.^20^

On the other hand, at high temperatures the heat exacerbates side reactions in the electrolyte and electrodes, which can speed up capacity fade and shorten the LIBs cycle life.^21,22^ When a LIB operates under overheating conditions, the increased reaction rates often lead to gas generation and internal pressure buildup; thus, raising the risk of venting, fire, or thermal runaway.^17,23^ Because of cost and current mass produced cell architectures, it is not possible to measure the temperature inside the battery—in between the electrodes where reactions happen.^24^ In addition to cost, the space inside the packs limits the installation of temperature sensors.^25^

Since direct measurement of internal cell temperature is often difficult in practice, thermal models are needed to capture heat generation and dissipation processes. These models provide a safer and more cost-effective way to predict and prevent unsafe operating conditions. In addition, the LIBs temperature correlates with state of charge (SOC) and state of health (SOH); therefore, the predicted temperature support high-accuracy in SOC and SOH assessments.^26^

The practical need for applications determines which thermal model should be used, since thermal modeling for LIBS can have different approaches.^27^ These models are typically divided into model-based approaches, such as equivalent-circuit and electrochemical–thermal models, and data-driven approaches, such as neural network (NN).^28^ Empirical and equivalent-circuit models are simple to implement; however, they do not take into consideration the internal chemistry or spatial temperature gradients.^28,29^ On the other hand, high-fidelity electrochemical models provide physics-based insight, yet usually involve heavy computation and many parameters that need to be determined.^28^ Purely data-driven methods can learn complex behavior of data; however, generally need large and carefully sampled datasets. Additionally, these models are commonly not effective in extrapolating to unseen conditions.^25,30^ Physics-informed neural networks (PINNs) have emerged as a promising alternative that uses small datasets and includes physics laws into the loss function to improve generalization.^31^

Although PINNs provide a way to integrate physics into data-driven models, some implementations may still rely on simplified heat terms. In particular, contributions such as entropy and radiative terms are not fully represented. While entropic heat is often overlooked or treated as a fixed average value, its impact have not been significant.^24,32–37^ For realistic thermal modeling, it is required to explicitly include the entropy contribution in the model equations.^37–41^ In addition, only a limited number of studies have investigated the role of thermal radiation in detail.^42,43^ At higher temperatures radiation may play a key role. For instance, Shadman Rad et al. showed that at around 40 °C, the radiative contribution to heat (HR) transfer can be greater than convection (HC) reaching 150% (HR/HC), while at lower temperatures it reduces to 46% (HR/HC).^39^

Concerning machine-learning strategies for battery state estimation and thermal modeling, Wang et al. proposed a Bayesian-optimized bidirectional LSTM for high-precision SOC estimation, while antinoise adaptive LSTM frameworks were introduced for robust remaining useful life prediction.^44,45^ More recently, electrochemical–thermal coupled models with real time parameter correction were developed for low-temperature operation.^46^ These approaches achieved strong predictive performance but rely primarily on data-driven learning or complex coupled models with online parameter identification. Previous efforts on temperature prediction of LIBs during operation have reached a mean square prediction error of 0.05 °C using 35% of the dataset.^25^ Such model only integrates Joule heating in PINNs and neglects entropic contributions, radiative losses, and the unit consistency of the adaptation of loss weights in the final loss function.^25^ The performance of this model was validated in a narrow thermal window, and the architecture presented computational complexity with the use of extra prelayers.^25^ Furthermore, the model was not validated for different cell chemistries and temperature conditions. In the work of Yuchen Wang, a model named BINN (battery informed neural network) was proposed for temperature prediction.^26^ Their approach reported mean square errors not higher than 0.150 °C (1C rate at 15 °C) for LFP cells tested across three different temperatures.^26^ The framework combines physics with machine learning, and integrates electrical, thermal, and heat transfer models, which allows estimation of hidden parameters such as heat capacity. However, the method relies on full life cycle data for training, requires heavy computation capacity, due to its complex structure, and was not validated near freezing temperatures, where safety issues are more critical.^26^

The main contributions of this work are summarized as follows: (i) an advanced PINN framework that comprises an adaptive coefficient ψ that dynamically balances the temperature, physics, and charge-consistency losses and make units consistent in the final loss function; (ii) an SOC-driven regularization term via a charge-consistency loss in the final loss function; and (iii) entropic heating and radiative contributions in the main physics equation (both within the governing thermal equation). Our model is validated using commercial cell data collected at 5 °C, 25 °C, and 45 °C for several cell chemistries, namely, lithium cobalt oxide (LCO), lithium iron phosphate (LFP), and lithium nickel manganese cobalt oxide (NMC). Importantly, a particular focus is given on low-temperature cycling (near freezing), an understudied area where conventional models often fall short.

## 2 Dataset Description

In this work, a dataset made available by Sandia National Laboratories was used to train and validate the proposed model across different chemistries and operating conditions.^47^ Each of the selected cells corresponds to a different widely adopted cell chemistry. As shown in Figure 1, the cell selection includes LCO (cell model LGDBHE21865, manufactured by LG Chem), LFP (cell model ANR18650M1, by A123 Systems), and NMC (cell model LGDBHG21865, by LG Chem). The main characteristics of the tested cells, including their chemistry, capacity, energy content, operating temperature range, and unit price, are summarized in Table 1. Each cell chemistry has distinct features that influence heat generation and thermal behavior. For example, the NMC cell delivers the highest energy (10.1 W h), whereas the LFP cell has the highest thermal stability and broadest operating temperature range (−30 to 60 °C).^48^ These different properties affect how the cells respond during charging and discharging and influence heat generation and dissipation during the cycling.

**Figure 1:**
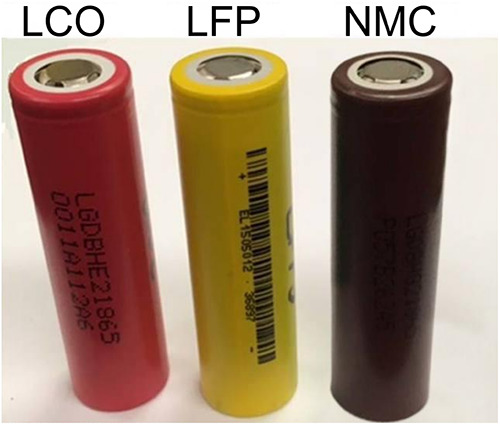
Commercial 18650-format lithium-ion cells from left to right: LGDBHE21865 (LCO), ANR18650M1 (LFP), and LGDBHG21865 (NMC). Adapted from Barkholtz et al.^47^ Copyright 2017 The Electrochemical Society.^47^

**Table 1:** Specifications of Various 18650-Format Lithium-Ion Cells Based on Cathode Material^47^

Property	LCO	LFP	NMC
Anode Material	Graphite	Graphite	Graphite
Cathode Material	LiCoO_2_	LiFePO_4_	LiNi_0.80_Mn_0.10_Co_0.10_O_2_
Nominal Capacity (Ah)	2.5	1.1	3.0
Nominal Voltage (V)	3.7	3.3	3.6
Energy (Wh)	8.8 ± 0.1	3.5 ± 0.02	10.1 ± 0.4
Energy Density (Wh/L)	533.3	212.1	612.1
Specific Energy (Wh/kg)	195.5	88.9	224.8
Discharge Current Max (A)	20	30	20
Operating Temp (°C)	0 to 50	–30 to 60	0 to 50
Volume (cm^3^)	16.5	16.5	16.5
Cell Mass (g)	44.9	39.4	44.9
Unit Price (USD)	9.95	9.95	6.00
Price per Energy (USD/kWh)	852.8	2,842.8	594.0

All cycling tests were performed using an Arbin SCTS multichannel battery testing system and carried out inside an SPX Tenney T10C-1.5 environmental chamber, which allows precise temperature control between −73 and 200 °C.^47^ Before cycling, cells were left at selected temperature for 12 h. While the cells were tested under various discharge conditions, only a portion of the cycling data corresponding to a 1C charge and 1C discharge rate were selected. To ensure consistency across all samples, the first step of the testing protocols was set to start at 0 h. The applied current profiles for each cell chemistry—NMC, LCO, and LFP—at 5 °C are shown in Figure 2(a–c). During the cycle, the cells were charged at a 1C rate using a constant current–constant voltage (CC–CV) protocol, where charging continued at constant voltage until the current dropped to below 50 mA. The cells were then discharged from full charge (100% SOC) to full 0% SOC also at 1C rate, with a rest period of 10 min between each charge and discharge cycle.

**Figure 2:**
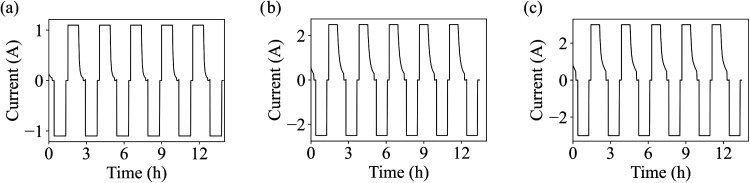
Current profiles for the three cell chemistries at 5 °C: (a) LFP, (b) LCO, and (c) NMC. Adapted from Barkholtz et al.^47^ Copyright 2017 The Electrochemical Society.^47^

For the cells selected, cycling tests were conducted at low (5 °C), room (25 °C), and high (45 °C) temperatures.^47^ Tests were stopped if the temperature of any cell exceeded the safe operating limit defined by the manufacturer (Table 1).^47^ At higher temperatures, particularly at (45 °C), self-heating effects could cause the environmental chamber to reach such cutoff threshold. When the threshold was reached, the system introduced automatic rest intervals, leading to segmented discharges. These pauses influenced the voltage behavior of the cells, which may cause some distortion in the voltage profiles. In order to monitor the temperature of the cell during cycling, a thermocouple was fixed to the outer surface of each cell. Generally, surface temperature measurements of the cell in battery research employ standard Type K or Type T thermocouples.^25,49–51^ According to commonly accepted specifications defined in ASTM E230/E230M, type K thermocouples in standard grade have limits of error of ±2.2 °C or ±0.75% of the reading (whichever is greater), while type T thermocouples provide better tolerances, with typical standard-grade limits of ±1.0 °C or ±0.75% of reading. Assuming the use of standard-grade thermocouples with typical surface-mounted configurations, the expected measurement uncertainty is approximately ±1 to ±2 °C, which accounts for factors such as thermal contact resistance, cold-junction compensation, and time lag.

In this work, the entropy-SOC relationship data was obtained from Wind and Vie, who used an accelerated potentiometric method to collect data at ≈5% SOC intervals across the full charge range and presented profiles for different chemistries.^52^ Regarding open-circuit voltage (OCV) curves for LFP and NMC chemistries, data was obtained from CALCE (Center for Advanced Life Cycle Engineering data—University of Maryland) database.^53–55^ However, for LCO cells, the OCV dataset collected was made available by a previous study on SOH assessment of commercial LIBs.^56^ Since both entropy and OCV measurements are conducted under equilibrium, small differences variations in the cell designs for the same chemistry—such as electrode loadings and N/P ratio—do not affect these measurements due to their thermodynamic nature.

## 3 Methodology

This section presents the methodology used to predict the surface temperature of LIBs during cycling. First, the general structure of the thermal model and the heat balance terms considered in this study are introduced, including radiative losses that are often neglected. Next, the proposed PINN framework is described, together with the way experimental signals are combined with governing thermal equations during training. The specific elements developed in this work are then identified, including the treatment of reversible entropic heat and the charge consistency constraint, and the adaptive weighting strategy that preserves physical units in the loss function. Finally, the network architecture, training procedure, and evaluation metrics are described to provide a clear and reproducible account of the approach.

### 3.1 Thermal Model

In the work of LeBel et al., it was demonstrated that including entropy-related heat in battery thermal models improves temperature prediction accuracy by up to 4 °C.^57^ The change in entropy (Δ*S*) describes how entropy differs between two states, especially during chemical reactions or physical processes such as lithium-ion intercalation and deintercalation in LIBs.^57^ Therefore, calculating entropy change at the electrodes reveals information about the battery’s energy efficiency and how its performance depends on temperature.^57^ While often neglected, this term can be endothermic or exothermic depending on the direction of the current and the SOC.^58,59^ The entropy contributions vary significantly across chemistries and SOC ranges, particularly in cells with sharp OCV–SOC transitions such as LCO.^52,58^ Including the entropy term helps capture these subtle but important thermal behaviors, especially at low-to-moderate C-rates and mid-SOC regions where reversible heat effects are more pronounced.^52,57,58,60^

In a temperature model, the Joule contribution term (*Q*_Joule_) accounts for the irreversible heat generated due to internal resistance during charge and discharge. As presented in eq 1, the *Q*_Joule_ component is directly linked to the current and the overpotential between the terminal voltage and the OCV. Since it reflects real-time electrical losses, it plays a central role in modeling temperature rise in LIBs under dynamic conditions.^61^ The heat generated by this mechanism dominates during high-rate cycling, especially when large current pulses are applied.^61^ Therefore, including this term in the thermal equation modeling ensures that energy losses are fully captured.^62^

1 $$Q_{\mathrm{Joule}}=I\left(V-\mathrm{OCV}\right)$$

where in eq 1, *I* is the current, *V* is the terminal voltage, OCV is the open-circuit voltage.

The radiative heat term (*Q*_rad_) accounts for the rate of energy losses through thermal radiation to the surrounding environment.^43^ Although *Q*_rad_—presented in eq 2—is often a small value at moderate temperatures, this term becomes more relevant at elevated operating conditions, where temperature gradients are larger. Radiative losses may influence the cell surface temperature prediction when modeling, especially during sustained high-temperature operation.^43^ From eq 2, it is possible to note that the radiative heat term becomes more significant when the difference between the *T* and (*T*_amb_) is large, since both temperatures are raised to the fourth power. Besides temperature, the cell’s surface emissivity (ε) also has a strong effect on this term. For this reason, accurate values of temperature and emissivity are important and should be carefully considered when building thermal models. Overall, including the *Q*_rad_ (eq 2) term improves the model’s fidelity at high ambient or self-heated temperatures and complements the convective loss term already present in eq 6. In addition, radiative heat transfer must be critically considered in LIBs thermal models because it plays a decisive role in overheating and failure. Studies have shown that radiation may dominate heat transfer at elevated temperatures, which strongly influences thermal runaway propagation between cells.^63^ Other investigations confirm that during failure events most of the heat exchange occurs through radiation.^64^ Ignoring this effect may lead to unsafe underestimation of heat flux, incomplete prediction, and catastrophic failure. Therefore, explicitly including the radiative term is necessary to ensure not only accurate an model, but also to improve the safety levels of LIB systems.

2 $$Q_{\mathrm{rad}}=\varepsilon \sigma A\left(T^{4}-T_{\mathrm{amb}}^{4}\right)$$

where in eq 2, ε is the cell emissivity, σ is the Stefan–Boltzmann constant, *A* is the cell surface area, *T* is the cell temperature, and *T*_amb_ is the ambient temperature.

Equation 3 describes the presence of a reversible heat source that results from changes in the entropy.^61,65^ The generation of entropic heat can be either endothermic or exothermic, depending on the direction of the electrode reactions and the SOC. The entropy change (Δ*S*) can be determined using the enthalpy change (d*H*) and the Gibbs free energy change,^39,66^ and after following additional derivations, this relationship can be expressed as shown in eq 4.^58^

3 $$Q_{\mathrm{entropy}}=T\Delta S\frac{I}{\mathrm{nF}}$$

4 $$\Delta S=\mathrm{nF}\left(\frac{\partial \mathrm{OCV}}{\partial T}\right)$$

where in eq 3, Δ*S* represents the change in entropy, (J mol^–1^ K^–1^), *n* refers to the number of electrons involved in the electrochemical reaction (consider to be equal to 1), and *F* is the Faraday constant.^58^

The entropy change can be experimentally measured via potentiometric or calorimetric techniques. However, these methods often require extended measurement periods. Therefore, to address this problem, alternative approaches that take less time, such as electrothermal impedance spectroscopy (ETIS) and an optimized protocol for potentiometric measurements can be employed.^67,68^ Once the entropy change is determined, the reversible heat power (*Q*_entropy_) can be calculated using eq 3.^37^ As presented in eq 5, *Q*_conv_ corresponds to the convective heat that represents the rate at which heat is transferred from the battery surface to the surrounding^69^

5 $$Q_{\mathrm{conv}}=\mathrm{hA}\left(T_{\mathrm{amb}}-T\right)$$

where in eq 5, *h* is the convective heat transfer coefficient. Our proposed model incorporates these key terms (eqs 1, 2, 3, and 5). The core of the proposed model is a physics-informed loss function that enforces the first-principles thermal behavior of the LIBs during operation. Specifically, the temperature dynamics is governed by the differential equation presented in eq 6

6 $$\frac{dT}{dt}=\frac{Q_{\mathrm{Joule}}+Q_{\mathrm{entropy}}-Q_{\mathrm{rad}}+Q_{\mathrm{conv}}}{mC_{p}}$$

where, in eq 6, *m* is the cell mass, and *C*_p_ is the specific heat capacity. Figure 3 illustrates the relative contributions of each term in eq 6 during operating conditions. Overall, the terms *Q*_Joule_ and *Q*_entropy_ correspond to the heat that arises from electrochemical processes inside the cell, whereas *Q*_conv_ and *Q*_rad_ terms describe heat removal from the cell surface to the environment.^70^ Together, these mechanisms form the thermal balance used in the governing equation for surface-temperature prediction.

**Figure 3:**
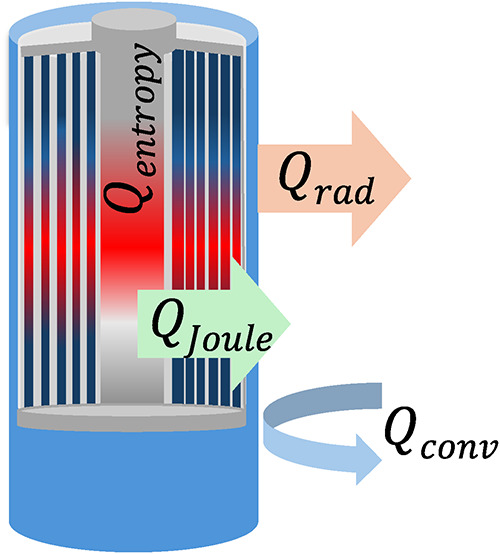
Thermal energy exchange in a LIB, showing contributions from Joule heating (*Q*_Joule_) and entropic heat generation (*Q*_entropy_), together with convective (*Q*_conv_) and radiative heat (*Q*_rad_) dissipation at the cell surface.

Concerning the charge term, the PINN predicts the cell temperature and charge at each time step. In this way, in order to obtain the charge via integration, the total charge delivered or stored by the LIB at each time step is computed by integrating the current over time

7 $$q\left(t\right)=\int_{0}^{t}I\left(\tau \right)d\tau$$

where eq 7 in discrete form is represented as

8 $$q_{i}=q_{i-1}+I_{i}\cdot \Delta t_{i}$$

where in eq 8, *I*_*i*_ is the current at step *i*, Δ*t*_*i*_ is the time interval, and *q*_*i*_ represents the total electric charge.

### 3.2 Enhanced PINN

In this work, the implemented enhanced PINN combines a data-driven network with thermal physics constraints (eq 6). The network receives as input time, current, voltage, charge, SOC, OCV, entropy coefficient, and temperature condition labels. Two outputs are generated: (i) the predicted temperature; and (ii) cumulative charge. A recurrent connection is introduced through the charge state to ensure temporal consistency.

The model was trained using a physics-informed loss, where the total loss function is composed of three terms: $L_{\mathrm{temp}}$ , $L_{\mathrm{phys}}$ , and $L_{\mathrm{charge}}$ . Each of these terms plays a specific role in constraining the predictions. The data term $L_{\mathrm{temp}}$ is the data mismatch term that ensures that the NN predictions follow the experimental measurements as closely as possible. It is defined as the mean squared error between predicted (*T* ^pred^) and measured (*T* ^exp^) temperatures, as represented in eq 9

9 $$L_{\mathrm{temp}}=\frac{1}{N}\sum_{i=1}^{N}{\left(T_{i}^{\mathrm{pred}}-T_{i}^{\exp}\right)}^{2}$$

The next term $\left(L_{\mathrm{phys}}\right)$ incorporates the heat balance equation directly into the training. It penalizes deviations between the predicted temperature dynamics and the thermal governing eq (eq 6). The physics loss is written as

10 $$L_{\mathrm{phys}}=\frac{1}{N}\sum_{i=1}^{N}r{\left(t_{i}\right)}^{2}$$

where in eq 10, the residual *r*(*t*) is expressed as

11 $$r\left(t\right)=\frac{dT^{\mathrm{pred}}}{dt}-\left(\lambda_{1}\left(Q_{\mathrm{Joule}}+Q_{\mathrm{entropy}}-Q_{\mathrm{rad}}\right)+\lambda_{2}\left(T_{\infty }-T^{\mathrm{pred}}\right)\right)$$

in eq 11, λ_1_ = (*mC*_p_)^−1^, and λ_2_ = *hA*/(*mC*_p_). By including $L_{\mathrm{phys}}$ , the model is guided to satisfy the underlying first-principles thermal equation rather than relying only on data fitting. The $L_{\mathrm{charge}}$ term enforces consistency in the cumulative charge prediction. The network predicts charge at each time step, which must match the value obtained from integrating the applied current. This constraint is expressed as

12 $$L_{\mathrm{charge}}=\frac{1}{N}\sum_{i=1}^{N}{\left(q_{i}^{\mathrm{pred}}-\left[q_{i}^{\mathrm{prev}}+\frac{I_{i}\Delta t}{3600}\right]\right)}^{2}$$

in eq 12, *q*_*i*_^prev^ is the cumulative charge predicted at the previous step, *I*_*i*_ is the applied current at time step *i*, and Δ*t* is the time increment (in seconds). As one of the contributions of the proposed model, this formulation enforces that the predicted charge trajectory remains consistent with the physical definition of charge as the time integral of current.

Since the terms for data, physics, and charge consistency operate on different scales, combining them directly in the total loss function may result in unbalanced training. If one loss dominates the other loss term, the model may either overfit or fail to satisfy physical constraints. To address this challenge, adaptive loss weights α and ψ are introduced so that each contribution has a comparable influence during training. As a result, using adaptive weights, the total loss used for training combining the three components $L_{\mathrm{temp}}$ , $L_{\mathrm{phys}}$ , and $L_{\mathrm{charge}}$ can be written as eq 13.

13 $$L_{\mathrm{total}}=L_{\mathrm{temp}}+\alpha \cdot L_{\mathrm{phys}}+\psi \cdot L_{\mathrm{charge}}$$

The weights are updated at each training step based on the gradients of the individual loss terms, namely $\nabla_{\theta }L_{\mathrm{temp}}$ , $\nabla_{\theta }L_{\mathrm{phys}}$ , and $\nabla_{\theta }L_{\mathrm{charge}}$ , where θ denotes the model parameters (weights and biases). In the update process, first the gradients of the individual loss terms are computed. The gradients are normalized and used to compute the raw weights.

14 $$\mathrm{\alpha ̂}=\frac{\max\left|\nabla_{\theta }L_{\mathrm{temp}}\right|}{\mathrm{mean}\left|\nabla_{\theta }L_{\mathrm{phys}}\right|+\epsilon }$$

15 $$\mathrm{\psi ̂}=\frac{\max\left|\nabla_{\theta }L_{\mathrm{phys}}\right|}{\mathrm{mean}\left|\nabla_{\theta }L_{\mathrm{charge}}\right|+\epsilon }$$

in the (eqs 14 and 15), $\max\left|\nabla_{\theta }L_{\mathrm{temp}}\right|$ and $\max\left|\nabla_{\theta }L_{\mathrm{phys}}\right|$ are the largest absolute gradients among all parameters (e.g., $\max\left(\left|\frac{\partial L}{\partial W_{1}}\right|,\left|\frac{\partial L}{\partial b_{1}}\right|,\cdot \cdot \cdot \right)$ ), while $\mathrm{mean}\left|\nabla_{\theta }L_{\mathrm{phys}}\right|$ and $\mathrm{mean}\left|\nabla_{\theta }L_{\mathrm{charge}}\right|$ denote the average absolute gradients across all parameters. A small constant ϵ is added to avoid division by zero. Finally, the weights are updated using an exponential moving average as expressed in

16 $$\alpha =\left(1-\gamma \right)\cdot \alpha_{\mathrm{prev}}+\eta \cdot \mathrm{\alpha ̂}$$

17 $$\psi =\left(1-\gamma \right)\cdot \psi_{\mathrm{prev}}+\eta \cdot \mathrm{\psi ̂}$$

where in (eqs 16 and 17), γ is the decay rate that controls the memory of previous values, η is the learning rate for adapting the weights, and α_prev_, ψ_prev_ are the weight values from the previous iteration.

In this process, the gradients of each loss with respect to the model parameters are first computed. Then, the relative sizes of these gradients are used to determine the suggested weights α̂ and ψ̂, which are then incorporated into the actual adaptive weights. This ensures that the influence of each loss term is dynamically balanced during training.

It is important to note that these adaptive weights are introduced to maintain consistency in the final loss function, which is often neglected in related studies. Because PINN loss functions combine data and physics terms that originate from different physical quantities, naive weighting strategies may lead to dimensional inconsistencies.^71^ This consistency is essential in PINN models to preserve a direct connection between the formulation and the underlying physics. The units of the three loss components follow directly from (eqs 9–12). From eq 9, the temperature mismatch term is

18 $$L_{\mathrm{temp}}=\left[K^{2}\right]$$

where in eq 18, *K* denotes Kelvin. In this section, units are indicated using square brackets. From (eqs 10 and 11), the residual *r*(*t*) represents the difference between two temperature

19 $$r\left(t\right)=\left[Ks^{-1}\right]$$

where in eq 19 *s* is the unit second. Therefore, the related loss unit is given by eq 20

20 $$L_{\mathrm{phys}}=\left[K^{2}s^{-2}\right]$$

The charge term in eq 12 is obtained from the time integration of current. The factor 1/3600 converts *I*Δ*t* from ampere-seconds to ampere-hours, which is the common unit used to express battery charge and capacity. Therefore, the charge variable is represented in the capacity domain [Ah]. As a result, the charge-consistency loss scales with the current magnitude, giving

21 $$L_{\mathrm{charge}}=\left[A^{2}s^{2}\right]$$

where *A* in eq 21 represents the unit ampere for electric current. The adaptive coefficients defined in (eqs 14 and 15) are obtained from ratios of gradient magnitudes. In practice, these gradients are evaluated numerically during backpropagation and therefore act as scaling factors. However, their magnitudes originate from loss terms associated with physical quantities. Therefore, the dimensional interpretation of the adaptive coefficients is derived from the units of the corresponding loss components rather than from the numerical gradients themselves. Considering the units of the associated loss terms, the ratios imply the following scaling for α (eq 22)­

22 $$\left[\alpha \right]∼\frac{\left[L_{\mathrm{temp}}\right]}{\left[L_{\mathrm{phys}}\right]}=\frac{K^{2}}{K^{2}s^{-2}}=\left[s^{2}\right]$$

To ensure consistent scaling in the charge-consistency term, the adaptive weight ψ is defined as ψ = ψ′ (Δ*t*)^2^, where ψ′ is obtained from the ratio of loss terms. This formulation arises naturally from the discrete-time representation of the charge update, where the integrated current is proportional to *I*Δ*t*. As a result, the charge-consistency loss introduces a time-squared dependence associated with the discretization. The scaling of ψ is inferred from the units of the loss terms, as shown in eq 23

23 $$\left[\psi \right]∼\frac{\left[L_{\mathrm{phys}}\right]}{\left[L_{\mathrm{charge}}\right]}=\frac{K^{2}s^{-2}}{A^{2}s^{2}}s^{2}=\left[\frac{K^{2}}{A^{2}s^{2}}\right]$$

Substituting these factors (eqs 22 and 23) into eq 13 gives

24 $$\alpha L_{\mathrm{phys}}=\left[s^{2}\right]\left[K^{2}s^{-2}\right]=\left[K^{2}\right]$$

25 $$\psi L_{\mathrm{charge}}=\left[\frac{K^{2}}{A^{2}s^{2}}\right]\left[A^{2}s^{2}\right]=\left[K^{2}\right]$$

Therefore, all contributions (eqs 18, 24, and 25) to the total loss share the same unit as presented in eq 26

26 $$L_{\mathrm{total}}=\left[K^{2}\right]$$

This shows that the adaptive weights provide a scaling that maintains dimensional consistency between the loss contributions in the total loss function.

#### 3.2.1 Model Architecture

Before training the model, all important features such as temperature, state of charge (SOC), current, and Voltage were normalized. This step ensures that each input feature is scaled to a common range (typically [0,1]), so that the NN can learn efficiently from data without being dominated by features with larger numerical values. For instance, the current normalization was calculated per dataset, using the absolute value of current, as shown in eq 27

27 $$I_{\mathrm{abs},\mathrm{norm}}=\frac{\left|I\right|-|I{|}_{\min}}{\left|I{|}_{\max}-\right|I{|}_{\min}}$$

Likewise, the other physical quantities such as time, Voltage, charge, and temperature were normalized using the general expression as follows

28 $$X_{\mathrm{norm}}=\frac{X-X_{\min}}{X_{\max}-X_{\min}}$$

where in eq 28, *X* refers to each feature (physical quantities).

The SOC was calculated as

29 $$\mathrm{SOC}=\frac{q}{q_{\mathrm{nominal}}}\times 100\%$$

from eq 29, *q* is the integrated charge (eq 7), and *q*_nominal_ is the nominal LIB capacity—given in Ah.

Several important features are derived from the raw data; therefore, interpolation was necessary. Interpolated OCV was carried so that the OCV voltage corresponding to each SOC was obtained via interpolation using a reference OCV-SOC lookup table, as described by eq 30

30 $$\mathrm{OC}V_{i}=\mathrm{Interp}\left(\mathrm{SO}C_{i},\mathrm{OCV}‐\mathrm{SOC}\;\mathrm{Table}\right)$$

In the same way, the interpolated entropy variation data (Δ*S* or d*S*), which impacts the entropy heat term, was interpolated as a function of SOC, defined in eq 31

31 $$dS_{i}=\mathrm{Interp}\left(\mathrm{SO}C_{i},\mathrm{Entropy}‐\mathrm{SOC}\;\mathrm{Table}\right)$$

Figure 4 shows the architecture of the proposed enhanced PINN model. The NN receives as inputs normalized time, current, Voltage, charge, SOC, OCV, entropy coefficient, and temperature condition label. Two outputs are generated, namely, the predicted temperature (*T*) and the cumulative charge (*q*). In each iteration, derivatives are calculated and $L_{\mathrm{total}}$ (eq 13) is then updated by combining the weighted contributions of the NN and the residuals from the physical equations. Typically, the training is carried out until $L_{\mathrm{total}}$ drops below a chosen tolerance (σ) at which the process is stopped. The training configuration is summarized in Table 2.

**Figure 4:**
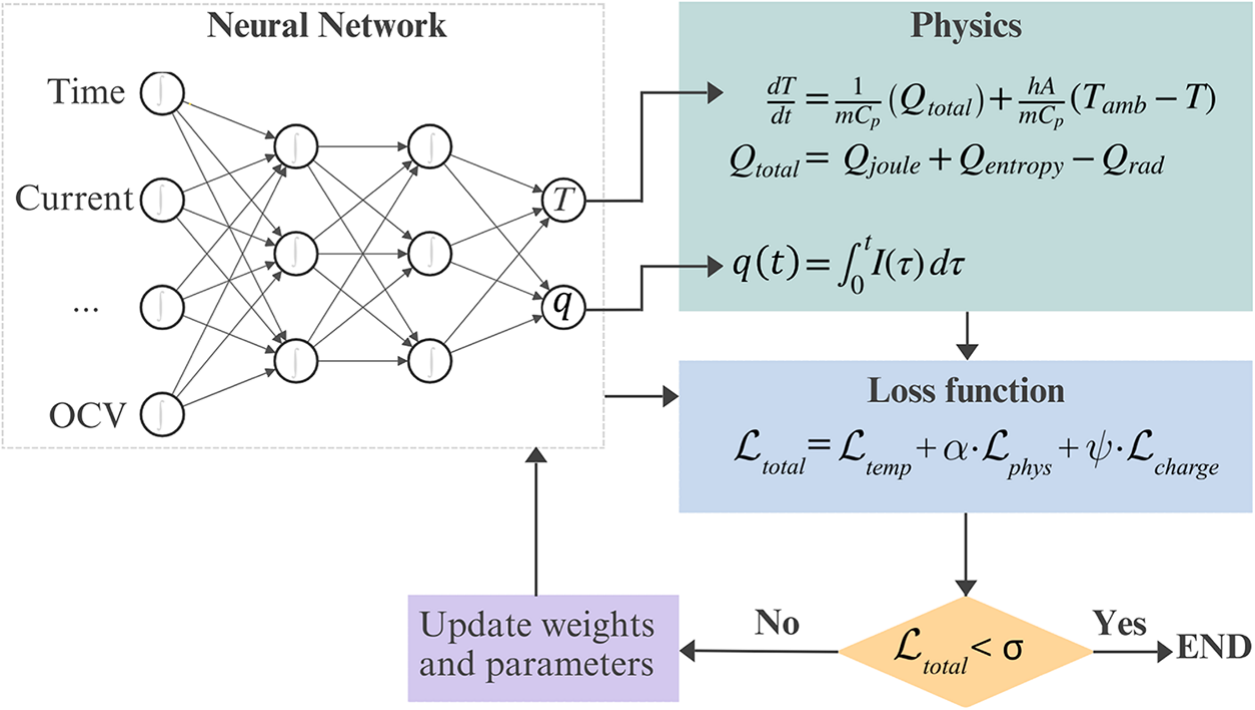
Schematic of the enhanced PINN architecture. The NN maps inputs to outputs, with physics-based regularization. The $L_{\mathrm{total}}$ function combines the weighted NN loss with the residuals of the governing equations. The training proceeds until the tolerance (σ) is satisfied.

**Table 2:** NN Hyperparameters Used in the Enhanced PINN Model

Hyperparameter	Value
Number of hidden layers	2
Maximum epochs	1500
Number of hidden neurons	128
Learning rate	1 × 10^–3^
Batch size	32
Trainable parameters	≈42,000

As presented in Table 2, the model employs two hidden layers with 128 neurons each, trained with the Adam optimizer using a learning rate of 10^–3^. Training was carried out for up to 1500 epochs with a batch size of 32, and the data were split into training and validation subsets. For training, 30% and 50% of each dataset were used for training to emphasize the generalization capability of the model. Overall, the network contains approximately 42,000 trainable parameters, which provides sufficient power to capture the nonlinear electrochemical–thermal interactions, while keeping the model computationally efficient for practical use. Additionally, the thermal parameters used in the governing equation used in the model, including heat capacity, convective coefficient, emissivity, and effective surface area, are summarized in Table 3. These parameters were not optimized or implicitly tuned during training. They were treated as fixed values in order to limit the number of free variables and focus the learning process on the governing physical formulation. The effective surface area (*A*), presented in Table 3, was estimated from the total external surface area of a 18650 cell (*A*_geometric_), adjusted by a utilization factor γ that accounts for transport-limitation phenomena under nonequilibrium conditions, such as varying temperatures and higher C-rates, as presented in eq 32

32 $$A=\gamma \times A_{\mathrm{geometric}}$$

where in eq 32, γ was estimated to be approximately 0.6, and *A*_geometric_ is equal to 0.0042 m^2^.^72^

**Table 3:** Thermal Parameters Used in the Governing Equation

Parameter	Value	Units	Refs
Specific heat capacity, *C*_p_	1100	J kg^–1^ °C^1–^	^73^
Convective coefficient, *h*	8.0	W m^–2^ °C^1–^	^74^
Emissivity, ε	0.9	–	^75^
Effective surface area, *A*	0.0025	m^2^	–

The model performance was assessed using mean absolute error (MAE) and root mean squared error (RMSE) between predicted and measured temperatures. The MAE is defined as eq 33

33 $$\mathrm{MAE}=\frac{1}{n}\sum_{i=1}^{n}\left|y_{i}-{ŷ}_{i}\right|$$

and the RMSE is given by eq 34

34 $$\mathrm{RMSE}=\sqrt{\frac{1}{n}\sum_{i=1}^{n}{\left(y_{i}-{ŷ}_{i}\right)}^{2}}$$

in (eqs 33 and 34), *y*_*i*_ is the measured value, *ŷ*_*i*_ is the value predicted by the model, and *n* is the total number of data points.

Both metrics were chosen for being commonly used in regression tasks to evaluate prediction accuracy.^76^ RMSE is more sensitive to outliers, emphasizing larger errors, and is suitable when errors follow a normal distribution. In contrast, MAE treats all errors equally and is less affected by outliers, providing a balanced error measure.^76^

### 3.3 Fully Connected Neural Network (FCN)

A fully connected neural network (FCN) was implemented to be used as baseline model as shown in Figure 5. According to Figure 5, the FCN network takes eight input features (time, current magnitude, current sign, Voltage, cycle time, temperature condition, OCV, and entropy coefficient) and outputs the predicted cell temperature.

**Figure 5:**
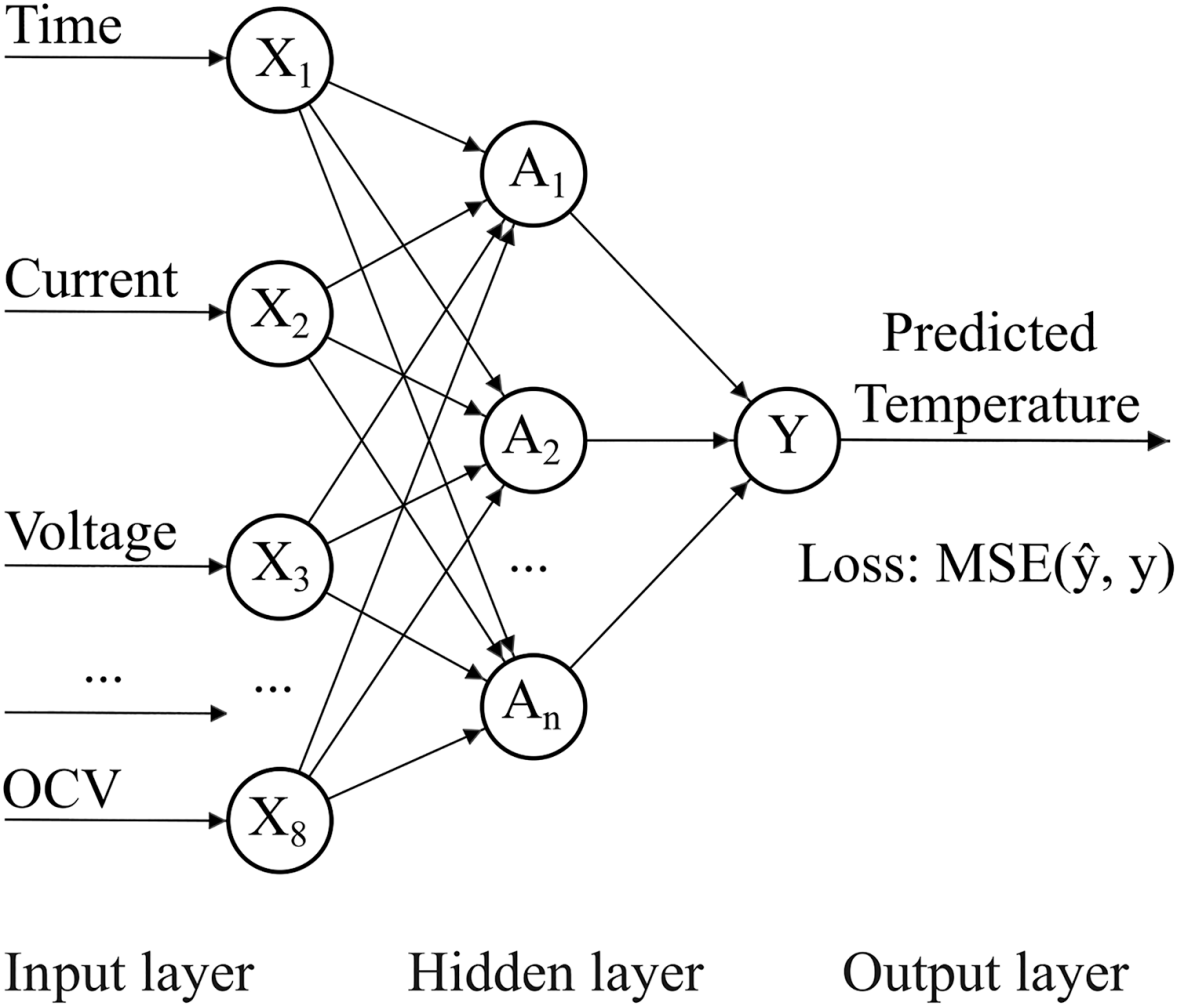
Structure of a back-propagation NN.

Learning occurs by minimizing the mean squared error between predicted and measured temperatures

35 $$L_{\mathrm{MSE}}=\frac{1}{N}\sum_{i=1}^{N}{\left(T_{i}^{\mathrm{pred}}-T_{i}^{\exp}\right)}^{2}$$

where in eq 35, *T*_*i*_^pred^ is the temperature predicted by the neural network for sample *i*, *T*_*i*_^exp^ is the corresponding measured temperature, and *N* is the total number of training samples. This loss function guides the model to reduce the difference between predicted and experimental temperatures, and learn the mapping from electrical and thermal inputs to the cell temperature.

The FNC model architecture consists of one input layer, followed by five hidden layers with 135–145 neurons each, using ELU as the activation function as presented in Table 4. Training was performed for 1500 epochs with a batch size of 32, and the Adam optimizer was applied with a learning rate of 10^–5^. In total, the network contains about 115,000 trainable parameters. This larger parameter count can provide capacity for learning nonlinear temperature responses, while the relatively small learning rate ensures stable convergence during training.

**Table 4:** NN Hyperparameters for the Baseline (FCN)

Hyperparameter	Value
Number of hidden layers	5
Hidden neurons per layer	135 (first), 145 (others)
Activation function	ELU
Maximum epochs	1500
Learning rate	1 × 10^–5^
Batch size	32
Trainable parameters	≈115,000

## 4 Results and Discussion

### 4.1 Low Temperature Performance Comparison for Different Cell Chemistries

The model performance comparison was conducted across three different chemistries. The validation was primarily conducted using cycling data cycled at 5 °C due to the challenges low temperature pose. The models were trained and evaluated separately for each chemistry-temperature pair. For each case, the dataset was split chronologically, using an initial continuous segment for training and a later segment for validation. This preserves the temporal structure of the data and ensures that training and validation data remain separate. Two training sizes of 30% and 50% were used, and the remaining portion of the selected dataset size was used for validation. When comparing 30% vs 50% training size, all settings are identical except for the length of the training window in each case. Detailed information regarding training and validation for 50% training size can be found in the Supporting Information (Figures S10–S18).

At 5 °C, the entropic heat (*Q*_entropy_) plays a more important role due to its dependence on SOC and the reversible nature of the process. Although the absolute temperature (*T*) is lower, variations in $\frac{dS}{dT}$ across the SOC range make *Q*_entropy_ non-negligible. In contrast, the radiative term (*Q*_rad_) shows less contribution at low temperatures, since the difference (*T* ^4^ – *T*_amb_^4^) remains small.^77,78^ The dominant cooling mechanism in this regime is the convective term $\frac{\mathrm{hA}}{{\mathrm{mC}}_{p}}\left(T_{\mathrm{amb}}-T\right)$ , which drives the cell temperature closer to the ambient temperature.

From the enhanced PINN model, the predicted temperatures considering a training dataset size of 30% model for LFP, LCO, and NMC are presented in Figures 6, 7 and 8, respectively. The respective errors are summarized in Table 5.

**Figure 6:**
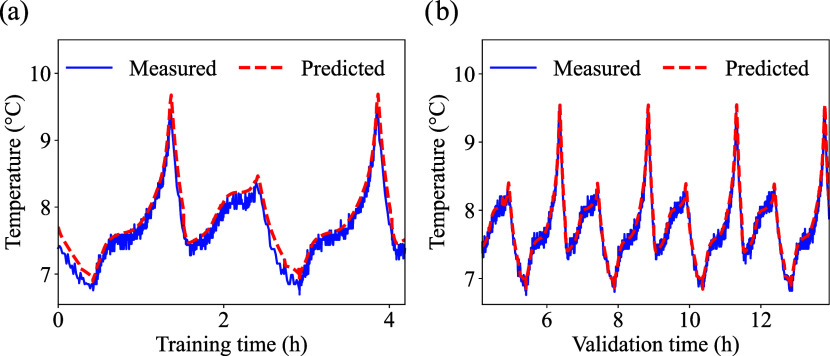
LFP cell low temperature prediction: (a) training, and (b) validation. The initial cell temperature was 5 °C.

**Figure 7:**
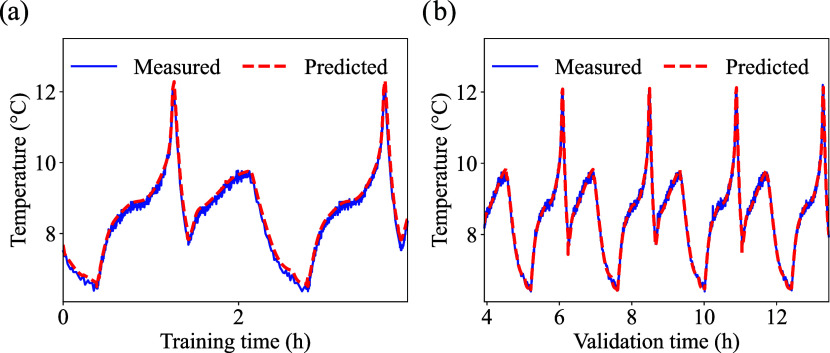
LCO cell low temperature prediction: (a) training, and (b) validation. The initial cell temperature was 5 °C.

**Figure 8:**
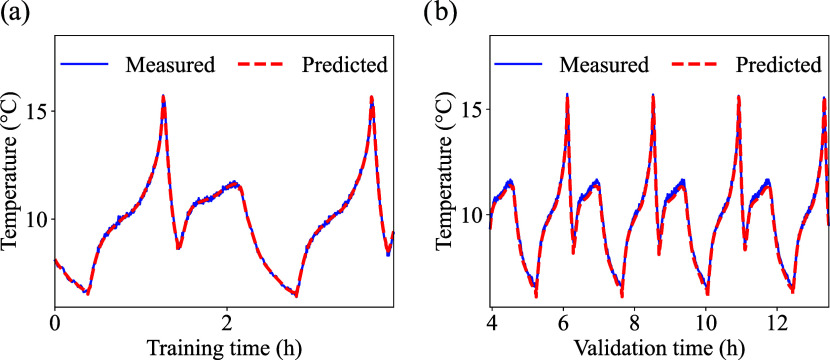
NMC cell low temperature prediction: (a) training, and (b) validation. The initial cell temperature was 5 °C.

**Table 5:** Temperature Prediction Errors at 5 °C for Different Battery Chemistries Using 30% and 50% of the Dataset for Training^a^

Training Size	Chemistry	Train MAE	Train RMSE	Val MAE	Val RMSE
30%	LFP	0.12	0.14	0.07	0.09
	NMC	0.06	0.08	0.13	0.16
	LCO	0.11	0.13	0.07	0.09
50%	LFP	0.05	0.07	0.08	0.10
	NMC	0.09	0.12	0.08	0.10
	LCO	0.06	0.08	0.10	0.12

a Errors are in °C.

Among the chemistries, the LFP cell exhibited the best performance during validation (Figure 6(b)), even with fewer data points used for training (Figure 6(a)). This suggests relatively stable and predictable thermal behavior under low temperature cycling. In addition, the accuracy performance, aligns with LFP’s flatter OCV–SOC shape, which makes entropy-driven variations easier for the model to learn. LFP’s OCV–SOC curve is mostly flat, presenting a plateau like feature.^79^ Therefore, $\frac{\partial \mathrm{OCV}}{\partial T}$ in eq 4 is nearly constant or zero in most of the SOC range and the entropy term adds little or very predictable heating. This way, the model does not have to learn sharp temperature shifts that are tied to SOC.

To understand how the size of the training data affects model performance, the results obtained using 50% of the dataset are also presented in Table 5. The temperature estimations for each chemistry using 50% of the training data show a close match between predicted and measured temperatures during both charge and discharge phases (Figures S10–S12). The model demonstrated strong predictive accuracy across all three chemistries: the validation MAE remained below 0.10 °C, and the RMSE did not exceed 0.12 °C. Notably, NMC matched LFP in validation MAE (0.10 °C), despite higher training error, which indicates good generalization of the model under similar conditions.

For training step using 30%, the improvements are more pronounced for the NMC chemistry (Figure 8(a)). While the OCV-SOC curve for LFP is mostly flat, the NMC and LCO OCV-SOC curve have more sloped and nonlinear profiles, which leads to greater variation in $\frac{\partial \mathrm{OCV}}{\partial T}$ across the SOC range.^80,81^ Since the PINN model includes Joule and entropy heat ((eqs 1 and 3), respectively), the cells with more complex $\frac{\partial \mathrm{OCV}}{\partial T}$ profiles (such as NMC) seems to benefit more from additional training samples to learn these variations and performer better during validation (Figure 8(b)).

Interestingly, reducing the training size from 50% to 30% led to a slight improvement in validation accuracy for LFP (Figure 6(b)) and LCO (Figure 7(b)) cells. In particular, for LCO the validation RMSE decreased from 0.12 to 0.09 °C and MAE from 0.10 to 0.07 °C (Table 5). A similar behavior is observed for LFP. This counterintuitive result may be related to the way the PINN regularization terms (particularly the charge-consistency constraint) help suppress overfitting when fewer data points are available. LCO cells exhibit sharp variations in entropy and heat generation at certain SOC ranges, which can amplify sensor noise and transient heating effects. However, with a shorter training window (for example Figure 7(a)), the model may focus on learning the dominant, smoother thermal trends rather than small local fluctuations, which results in slightly better generalization. These findings highlight that the enhanced PINN may benefit even to a small extent, when trained on reduced datasets, especially for chemistries with stronger SOC-dependent thermal dynamics.

It is important to note that the reported MAE values must be interpreted in the context of measurement uncertainty. The surface temperature was measured using a thermocouple with an expected uncertainty of approximately ± 1–2 °C. No filtering or smoothing was applied to the temperature signals prior to training. Therefore, the model was trained directly on the raw measured data. Additionally, the thermocouple uncertainty defines a practical lower bound (error floor) for absolute temperature accuracy. The reported validation MAE values (as low as 0.07 °C) are significantly smaller than the sensor uncertainty and indicate that the model reproduces the measured trajectory within the experimental noise level. However, this does not imply physical accuracy beyond the intrinsic measurement limits. Consequently, the improvements should be interpreted as improved consistency with the measured signal rather than enhanced absolute thermodynamic precision. In this context, the analysis focuses on deterministic prediction accuracy relative to the measured signal. A rigorous statistical uncertainty analysis would require additional experimental data beyond the dataset currently available from the public Sandia National Laboratories database used in this study.

LIBs can experience temperature changes due to external conditions or heat generated during its use.^82–84^ These effects become more noticeable at higher C-rates, which influence ion flow and increase *Q*_Joule_.^82,84,85^ In particular, during low SOC operation, heat tends to build up in specific areas of batteries. This heat concentration is likely due to the lack of active material and lower reaction activity in low-SOC conditions, which may reduce overall heat generation that may lead to uneven temperature distribution and local thermal variations.^86^ However, it is possible to note from Figures 6–8(a,b) that the model accurately follows the measured temperature trends during cycling, highlighting the potential contribution of the $L_{\mathrm{charge}}$ term that helps regulate these SOC-dependent heating effects. In addition, for all temperatures, external factors such as electromagnetic interference can also affect K- and T-types thermocouple readings, and therefore reduce battery model accuracy.^87^

These results confirm that the proposed enhanced PINN model is capable of learning temperature dynamics effectively in low-temperature conditions, where battery thermal responses are typically challenging to model.^20,88,89^ While the findings indicate that more training data (50% of the dataset) help the model to capture nonlinearities better (Figures S10–S12), the results also demonstrate that the PINN approach maintains strong performance even when trained on a smaller portion (30%) of the dataset.

To assess the performance of the proposed PINN approach, a comparison was carried out against a standard FCN using the 30% training dataset. The FCN represents a traditional neural network, where the loss function is defined only by the mean squared error between predictions and measured data. The FCN‘s architecture consists of hidden layers connecting the input variables (time, current, voltage, and OCV) to the output variable (cell temperature). The comparison was conducted across three different chemistries for the same dataset. Results of cells cycled at low temperature are presented in Figures 9, 10, and 11. More information regarding the performance of the FCN model for different temperatures and chemistries can be found in the Supporting Information (Table S1). For all curves, the training size of 30% used for the training (Figures 9(a), 10(a) and 11(a)), and the remaining portion of the selected dataset size was used for validation (Figures 9(b), 10(b) and 11(b)). As mentioned previously, the PINN and the FCN baseline share the exact same training/validation splits.

**Figure 9:**
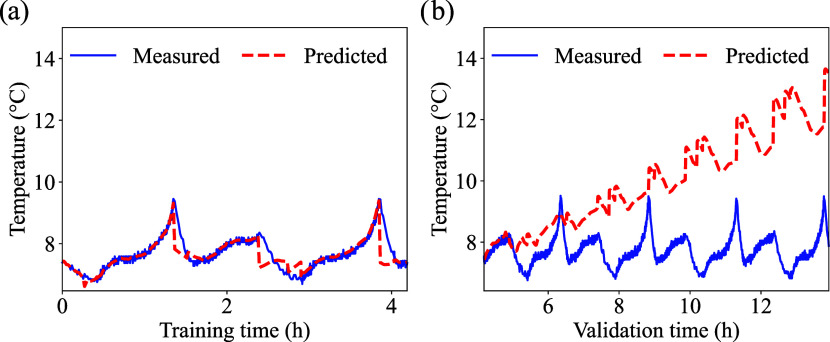
FNC - LFP cell low temperature prediction: (a) training, and (b) validation. The initial cell temperature was 5 °C.

**Figure 10:**
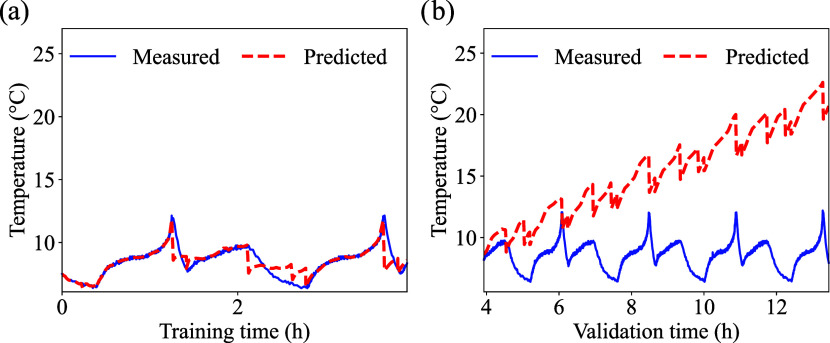
FNC - LCO cell low temperature prediction: (a) training, and (b) validation. The initial cell temperature was 5 °C.

**Figure 11:**
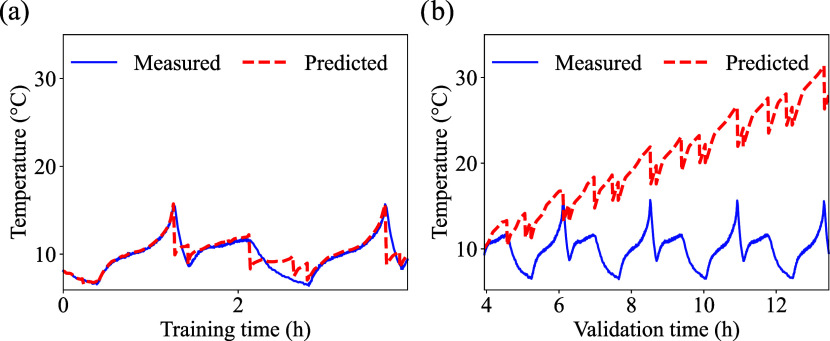
FNC - NMC cell low temperature prediction: (a) training, and (b) validation. The initial cell temperature was 5 °C.

Although the FCN model is able to reproduce the general profile of the temperature curves, a clear drift in the predicted values is observed over time—Figures 9(b), 10(b) and 11(b) for LFP, LCO, and NMC, respectively. For all cases (Figures 9(b), 10(b) and 11(b)), the predicted temperatures continue to increase compared to measured temperatures even when the measured profile stabilizes. This systematic deviation may occur because the FCN relies only on data fitting and lacks physical constraints that would limit the temperature rise. Since time is one of the main input features, the network may associate longer cycling periods with higher temperatures, leading to an artificial upward trend. This behavior highlights the limitation of purely data-driven models in capturing realistic thermal dynamics of batteries, particularly when the true response involves saturation effects or steady-state conditions.

The performance of the FCN is summarized in Table 6. According to Table 6, the FCN model shows clear limitations when predicting cell temperature at low ambient conditions. The validation errors are considerably high across the three chemistries, particularly for NMC and LCO cells. For instance, the validation MAE exhibited 9.99 °C for NMC and 6.69 °C for LCO, clearly indicating large deviations from the measured values. Only the LFP cell achieved relatively lower errors, with a validation MAE of 2.16 °C; however, this value still exceeds the typical uncertainty of thermocouple measurements (1 to 2 °C). These results confirm that the FCN struggles to capture the complex electrochemical–thermal behavior of lithium-ion cells at low temperature when limited training data are available.

**Table 6:** FNC Performance for Different Battery Chemistries Cycled at 5 °C Using 30% of the Dataset for Training^a^

Chemistry	Train MAE	Train RMSE	Val MAE	Val RMSE
NMC	0.35	0.58	9.99	11.34
LFP	0.09	0.18	2.16	2.64
LCO	0.17	0.35	6.69	7.56

a Errors are in °C.

Results presented in Tables 5 and 6 show clear differences between the FCN and the proposed enhanced PINN. For the FCN, the validation errors were high, with values up to 9.99 °C for NMC and 6.69 °C for LCO. In contrast, the PINN reduced the error to below 0.13 °C for all chemistries, including 0.07 °C for both LFP and LCO. The PINN errors are more than 70 times smaller than those of the FCN in some cases. This difference arises because the PINN integrates physical component constraints that guide the learning process, which allows the PINN to follow both the rise and the stabilization of the temperature curve.

#### 4.1.1 Performance Comparison for Intermediate (25 °C) and Higher (45 °C) Temperatures for Different Cell Chemistries

At 25 °C, the evolution of the cells’ temperature results from a combination of Joule heating (*Q*_Joule_), reversible heat (*Q*_entropy_), and ambient interactions. Although radiative heat loss (*Q*_rad_) is less significant at this temperature, the contribution of entropy heat increases. The errors using 30% and 50% of the dataset are summarized in Table 7. In general, for all cells when trained on 30% of the data, the validation MAEs remained below 0.15 °C.

**Table 7:** Enhanced PINN Temperature Prediction Errors at 25 °C for Different Battery Chemistries Using 30% and 50% of the Dataset for Training^a^

Training Size	Chemistry	Train MAE	Train RMSE	Val MAE	Val RMSE
30%	LFP	0.04	0.05	0.06	0.07
	NMC	0.07	0.09	0.14	0.19
	LCO	0.07	0.08	0.07	0.10
50%	LFP	0.04	0.05	0.05	0.06
	NMC	0.08	0.10	0.10	0.12
	LCO	0.06	0.08	0.06	0.07

a Errors are in °C.

The LFP cell showed the most stable results, with a validation MAE of 0.06 °C when trained on 30% and only a slight decrease to 0.05 °C with 50% training. At 25 °C, this performance is also attributed to LFP’s flat OCV-SOC profile, which results in a nearly constant ∂ OCV/∂ *T*. This stability reduces the complexity of modeling entropy heat (eq 3), making the behavior less difficult for the PINN to learn.

As shown in Table 7, the prediction errors for the LCO cell remain low across both training conditions, with validation MAE values of 0.07 and 0.06 °C when using 30% and 50% of the dataset, respectively. At 25 °C, the variation in temperature during cycling results from a combination of Joule heating (*Q*_Joule_) and entropy-related heating (*Q*_entropy_), both of which depend on ∂ OCV/∂ *T*. This effect is more pronounced in chemistries such as LCO, where the entropy contribution is larger and the OCV-SOC profile exhibits sharper transitions.^52^ It is important to note that the adaptive weight ψ, that is associated with charge-consistency in the loss function, helps the model capture SOC dynamics around room temperature. This becomes more important when SOC-dependent effects (such as *Q*_entropy_) has considerable contribution. Therefore, the inclusion of this term improves prediction stability even with limited training data, as displayed by LCO. In contrast, NMC-based cells exhibit lower entropy-SOC variation, which makes the cells less sensitive to entropy coefficient changes.^52^ Interestingly, NMC shows the highest validation error when trained on only 30% of the data (Val MAE: 0.14 °C), but this error exhibits only a slight decrease to 0.10 °C with 50% training of the total dataset. This small improvement in the accuracy, may indicate that the model benefits from additional data points in the training, which is likely due to better coverage of nonlinearities. Despite not being cycled in lower temperature conditions, the localized heat in the cell can still occurs during low SOC operation. This heating is associated with reduced electrochemical activity and limited active material available in these regions of the cell, which can lead to uneven heat generation.^86^ Therefore, during temperature measurement, this process can cause noisy data and reduce the overall precision of the model.

The stable predictions across different chemistries, around room temperature, suggest that the model learns the underlying thermal behavior driven by SOC changes. In particular, the adaptive charge-consistency ψ allows the model to better capture subtle variations related to entropy, which can be difficult to model purely from data. This makes the approach particularly valuable for cell chemistries with more complex entropy-SOC profiles.

At 45 °C, the cells operate under elevated thermal stress.^90^ For safety purposes, during cycling, the tests were stopped when the cells reached the maximum temperature allowed by the manufacturer (Table 1). At high temperatures, the battery’s self-heating are more significant, and caused the thermal chamber to reach its cutoff limit of 50 °C. For this reason, The discharge process was interrupted by rest periods which reflects on the voltage profile and the thermal response of the cells.

The temperature prediction errors for cells cycled at 45 °C are summarized in Table 8. From Figures S7–S9, it can be observed that the estimated curves track the behavior of the measured temperature curve closely across all chemistries.

**Table 8:** Enhanced PINN Temperature Prediction Errors at 45 °C for Different Battery Chemistries Using 30% and 50% of the Dataset for Training^a^

Training Size	Chemistry	Train MAE	Train RMSE	Val MAE	Val RMSE
30%	LFP	0.05	0.06	0.06	0.09
	NMC	0.05	0.07	0.07	0.09
	LCO	0.05	0.07	0.07	0.10
50%	LFP	0.05	0.06	0.06	0.08
	NMC	0.05	0.06	0.07	0.09
	LCO	0.05	0.06	0.05	0.07

a Errors are in °C.

According to Table 8, validation MAE values were generally within 0.06 to 0.07 °C using 30% of the dataset. Overall, compared to the 50%, the validation errors for the 30% training set were close. Similar behavior is observed during traing step. This small difference, supports the efficient trade-off between training size and model accuracy for cells that were also cycled at higher temperature. In general, the model performed best at 45 °C compared to the cells cycled at 5 °C (Table 5) and 25 °C (Table 7). This performance is likely due to the high-temperature effects, such as improved electrolyte conductivity and faster reaction kinetics, that can make the cell thermal behavior more consistent and less sensitive to small fluctuations.^91–93^ Additionally, for higher temperatures there is a reduction of the relative contribution of the entropy term (*Q*_entropy_). Instead, as the *Q*_Joule_ heating becomes dominant, which is directly related to current and voltage difference *I*(*V* – OCV), both of which are directly input into the PINN. The direct imputing of these data can also be associated with the improved performance at higher temperatures.

### 4.2 Additional Validation and Robustness Studies

Additional validation and robustness studies were conducted using a representative test case. These analyses assess the sensitivity of the model to key physical parameters, its ability to generalize across operating conditions and cell chemistries, and the role of selected physical terms in limiting extreme temperature errors.

A sensitivity analysis was conducted for LCO cells, which were selected as a representative case. The effective surface area was increased from 0.0025 m^2^ to 0.0042 m^2^ (approximately 70%) while all other parameters and training conditions were kept unchanged. At 5 °C, validation errors remained unchanged by increasing the effective surface area, which shows that the model is strong to access low temperatures. At higher temperature, the largest validation RMSE observed was 0.23 °C (Table S2). This behavior is physically consistent, since both convective and radiative heat-transfer terms scale with surface area and become more influential at elevated temperatures. These results indicate that the model responds to realistic parameter variations while maintaining low error levels in most operating regimes.

To further assess performance outside the training regime, cross-condition evaluations were conducted using LCO cells for training and LFP and NMC cells for validation at 5 °C, 25 °C, and 45 °C. The same training split was used in all cases, consisting of 3.9–4.7 h of recorded LCO data at each temperature (30% of the available dataset). For validation, the complete datasets of the unseen chemistries were used, covering 12.9–14.1 h. At 45 °C, the best transfer case was obtained when training on LCO and validating on NMC, resulting in a validation MAE of 0.29 °C and RMSE of 0.36 °C (Table S3). At 5 °C, the lowest errors were observed when training on LCO and validating on LFP, with a MAE of 1.55 °C and RMSE of 1.72 °C. Overall, the results show that performance decreases when the model is applied to different chemistries, particularly at low temperature. The differences in electrode materials, entropy behavior, and OCV–SOC relationships across chemistries introduce additional variability that can reduce predictive accuracy. However, the present framework is mainly intended for interpolation within a given chemistry and operating regime. Therefore, for real-world deployment, retraining with chemistry-specific data would be needed to achieve comparable accuracy when applying the model to new cell types.

A complementary ablation analysis was performed for LCO cells by reporting the maximum local temperature error over the validation period, defined as the largest difference between measured and predicted temperature profiles. This metric is relevant for battery management because brief temperature peaks can determine safe operating limits and trigger protective control actions. Using 30% of the data for training, the full model reached maximum errors of 0.74 °C at 5 and 0.64 °C at 45 °C (Table S4). For instance, at high temperature, removing either physical contribution led to larger peak deviations, with maximum errors increasing to 1.13 °C without radiative losses and 1.30 °C without entropic heating. These results highlight that including both physical terms can reduce large temperature prediction errors at elevated temperature. This is critical for thermal control and reliable battery operation, including improved detection of overheating conditions.

When using 30% of the dataset, for all temperatures, the model achieved errors that are below the typical uncertainty range of thermocouple-based measurements. This suggests that the model predictions are at least as consistent as the sensor readings under the tested conditions. Although the model presents good performance across different chemistries, the characteristics of LFP cells used make the measured signals inherently noisier. However, the model analysis also provides useful insight into the reasons behind this behavior, which can be attributed to a combination of intrinsic and experimental factors. The LFP cell presents lower heat generation that originates from the smaller nominal capacity and specific energy compared to NMC and LCO (Table 1). These characteristics results in smaller thermal gradients that can make the signal variations less pronounced during the measurement. The fluctuations that would normally be negligible for chemistries with steeper OCV–SOC curves can become more pronounced in LFP, which can give the measured data a noisier appearance. The nearly flat OCV–SOC profile of LFP reduces the variation of $\frac{\partial \mathrm{OCV}}{\partial T}$ across most of the SOC range. Under these conditions, even small errors in voltage measurement or SOC estimation translate into disproportional large changes in the calculated entropy-related heat. Additionally, the surface-mounted thermocouple configuration, the limitations such as ±1–2 °C uncertainty, contact resistance, and electromagnetic noise can further influence the effect of noise that comes from measurement.

Since surface-mounted thermocouples typically exhibit uncertainties on the order of ±1 °C, prediction performance was also evaluated relative to sensor tolerance. For all chemistries and ambient temperatures, 30% of each dataset was used for training and the remaining data for validation. The fraction of validation samples within a ±1 °C band was therefore computed for the proposed model. Across all cases considered, 100% of validation points fell within this tolerance, indicating strong consistency with the measured surface temperature signals. Accordingly, sub-±0.1 °C mean errors are framed as agreement with experimental data rather than as resolution beyond the limits of the sensing hardware. This confirms that the proposed model tracks experimental thermal behavior reliably within measurement uncertainty, which is valuable for battery management and safety-related applications.

It is important to note that the present model predicts surface temperature rather than internal electrode or core temperatures. While surface thermal behavior can provide useful indirect information for battery management, internal temperature gradients and localized heating would require additional modeling or internal temperature sensors. Therefore, the present framework is intended to support surface-based monitoring and control, rather than direct diagnosis of internal degradation mechanisms, such as lithium plating and thermal runaway.

### 4.3 Future Research Directions

The validation presented in this work was performed using single commercial 18650 cells cycled at a 1C rate under controlled environmental chamber conditions. Surface temperature was measured using surface-mounted thermocouples. These conditions allow the thermal behavior of the cells to be analyzed in a controlled setting. However, real battery systems may experience additional effects that were not considered in this study. For example, pack-level thermal coupling, cell unbalancing, nonuniform airflow, sensor placement variability, and aging processes may influence the observed temperature response. In addition, batteries operating in practical applications are often subject to more dynamic load profiles and changing environmental conditions. Therefore, extending the proposed framework to pack-level configurations and more complex operating scenarios is an important direction for future research.

Another limitation concerns the evaluation metrics. The present study evaluates model performance using deterministic prediction metrics. However, additional data beyond the public Sandia National Laboratories dataset used in this study would be required to perform a rigorous statistical uncertainty analysis. For this reason, the evaluation focuses on deterministic error metrics such as MAE and RMSE, together with validation across operating conditions and sensitivity tests. Future studies may explore uncertainty quantification methods, such as ensemble approaches or Bayesian frameworks, using datasets that include more repeated experiments. Additionally, future work will focus on modeling temperature-dependent parameters such as *h* and *C*_p_, and extending the approach to more dynamic scenarios, including eVTOL mission and electric vehicle driving profiles, and broader datasets.

## 5 Conclusion

In this work, we present an enhanced PINN architecture for battery surface-temperature prediction. The proposed model embeds the governing thermal equations within the training process and explicitly includes entropic heating and radiative losses, which are often neglected in prior studies. A charge-consistency term is also introduced to enforce SOC-dependent dynamics. In addition, adaptive coefficients α̂ and ψ̂ are used to balance the physical and data-driven loss terms during training. These coefficients are defined to ensure unit consistency in the final loss function, which is frequently overlooked in related work. Such consistency is important and expected in PINN models to maintain a clear link between the formulation and the underlying physics. The model also demonstrates adaptability across multiple chemistries (LFP, LCO, NMC) and operating conditions (5 °C, 25 °C, and 45 °C). This study findings outline that the enhanced PINN model is effective even with limited training data (30%), unlike purely data-driven models. At 5 °C, the proposed model achieves validation errors in general below 0.10 °C when trained with only 30% of the dataset. This performance was comparable to the case of 50% training dataset. On the other hand, a traditional FCN model deviated by several degrees under the same conditions. These results unveil that the proposed enhanced PINN model is capable of reliably capturing the main thermal dynamics of cells using limited data even under low-temperature conditions—despite all challenges lower temperature prediction pose. When using the limited training data (30%), the charge-consistency loss effectively regulates SOC-dependent heating effects, which is especially important at low temperatures. To enhance physical reliability, the adaptive loss balancing (α and ψ) ensures that Joule, entropic, and radiative contributions are appropriately weighted. Consequently, the model also generalizes well at high temperatures. In these scenarios, Joule heating dominated the thermal response, while entropic contributions were less significant. Overall, performance was highest at elevated temperatures, with validation MAE in the range 0.05–0.07 °C, which makes the enhanced PINN model also suitable for applications such as preventing battery overheating. In practice, the prediction errors reported in this work are comparable to the expected uncertainty of surface-mounted thermocouples (approximately ±1–2 °C). As a result, the proposed model shows consistent agreement with experimentally measured temperature signals across different chemistries and testing conditions, supporting its use for monitoring and control in battery management systems. This study presents the first PINN model that effectively integrates relevant physical terms, leading to more reliable and robust battery system predictions. In addition, this work places emphasis on low temperature behavior, addressing a critical yet understudied topic in battery performance and safety.

## Supplementary Material



## Notes

Extended current profiles and temperature prediction results across chemistries and temperatures at 5 °C, 25 °C, and 45 °C (Figures S1–S18); and baseline model comparison, sensitivity analysis, cross validation results, local error for LCO cells, and conceptual model comparison (Tables S1–S5) (PDF)
