# Supplementary material for: Low-Temperature Prediction in Commercial Lithium-Ion Batteries during Dynamic Usage via Enhanced Physics-Informed Neural Networks
Source: ACS Omega. 2026 May 13;11(20):29461–76. doi: 10.1021/acsomega.5c11152 (PMC13216964; doi:10.1021/acsomega.5c11152)
Supplement: Supplementary file 1 [file ao5c11152_si_001.pdf]

Supplementary information

Low-Temperature Prediction in Commercial Lithium-Ion Batteries During Dynamic Usage via Enhanced Physics-Informed Neural Networks

Eric L. Pereira [a*], Davi M. Soares [a]

[a]Department of Electrical and Computer Engineering
Wichita State University, 1845 Fairmount Street,
Wichita, KS 67260, United States

* Email: expereira@shockers.wichita.edu

1 Applied current profiles

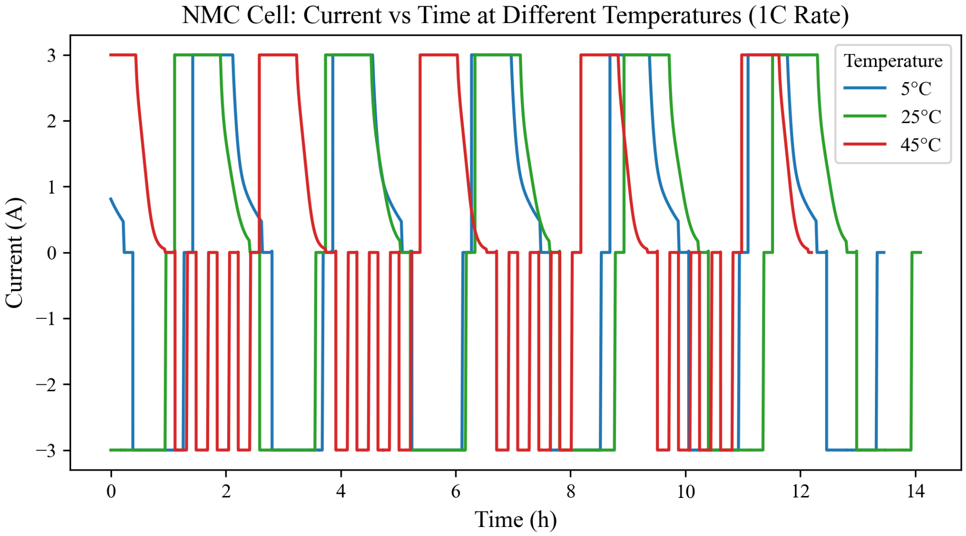

Figure S1: Current versus time profile for the NMC cell (LGDBHG21865) cycled at 1C under three different temperatures: 5 °C, 25 °C, and 45 °C. Adapted from Barkholtz et al., A Database for Comparative Electrochemical Performance of Commercial 18650 Format Lithium Ion Cells, Journal of the Electrochemical Society, 2017, DOI 10.1149/2.1701712jes. Copyright 2017 The Electrochemical Society [1].

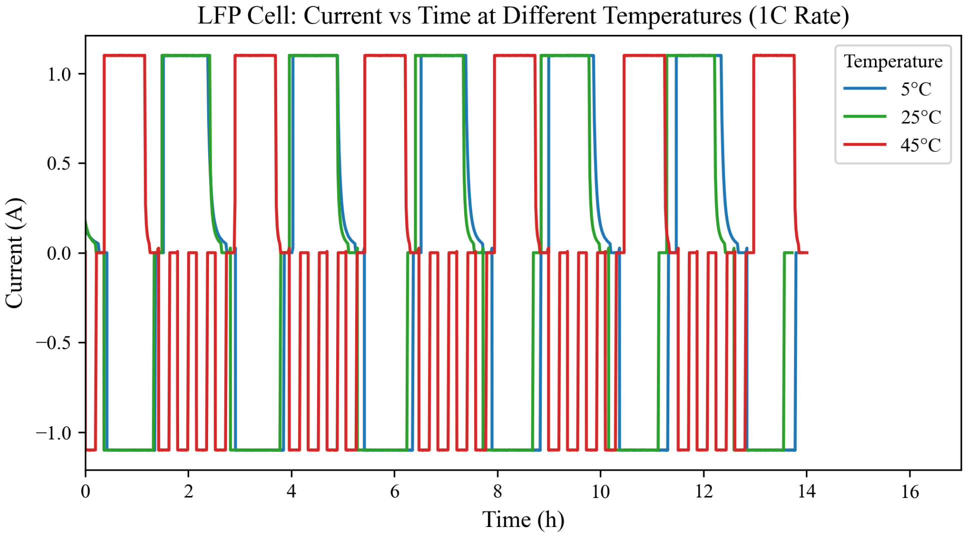

Figure S2: Current versus time profile for the LFP cell (ANR18650M1) cycled at 1C under three different temperatures: 5 °C, 25 °C, and 45 °C. Adapted from Barkholtz et al., A Database for Comparative Electrochemical Performance of Commercial 18650 Format Lithium Ion Cells, Journal of the Electrochemical Society, 2017, DOI 10.1149/2.1701712jes. Copyright 2017 The Electrochemical Society [1].

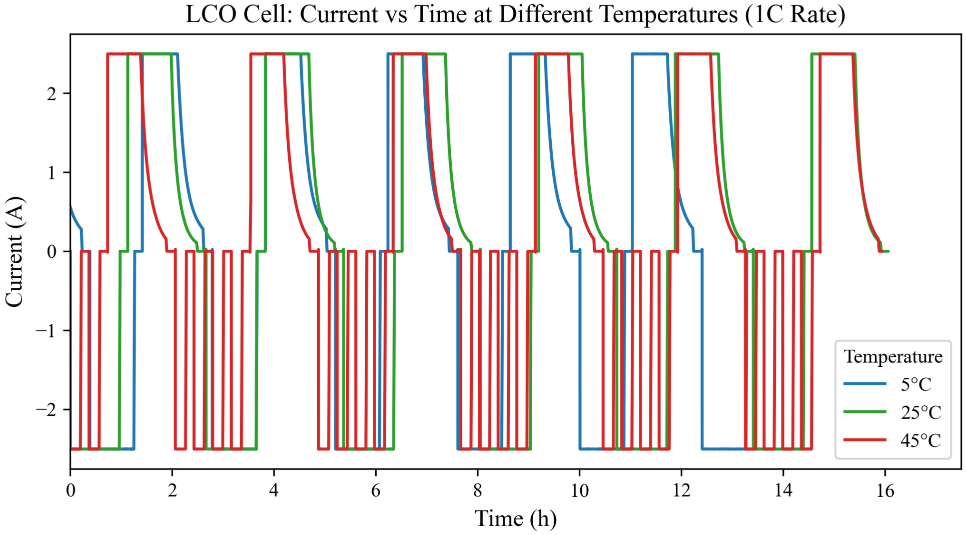

Figure S3: Current versus time profile for the LCO cell (LGDBHE21865) cycled at 1C under three different temperatures: 5 °C, 25 °C, and 45 °C.Adapted from Barkholtz et al., A Database for Comparative Electrochemical Performance of Commercial 18650 Format Lithium Ion Cells, Journal of the Electrochemical Society, 2017, DOI 10.1149/2.1701712jes. Copyright 2017 The Electrochemical Society [1].

2 Temperature predictions using 30% of the dataset

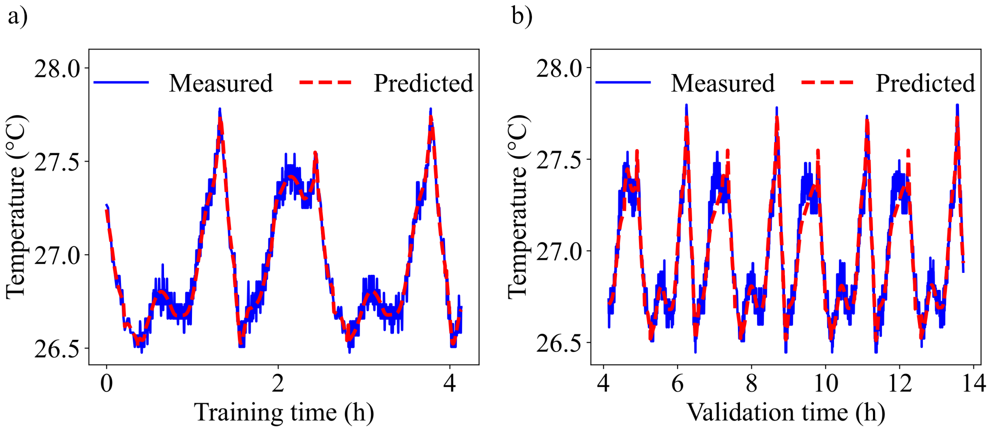

Figure S4: LFP cell around room temperature prediction: (a) training, and (b) validation. The initial cell temperature was 25 °C.

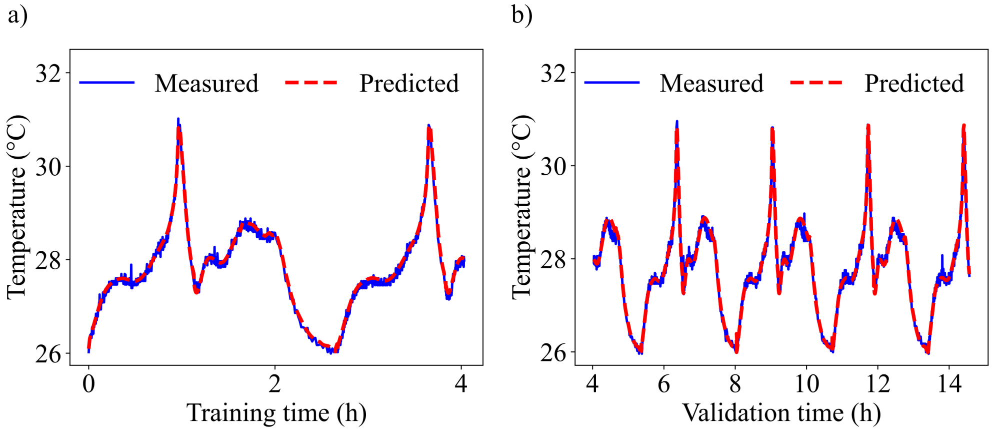

Figure S5: LCO cell around room temperature prediction: (a) training, and (b) validation. The initial cell temperature was 25 °C.

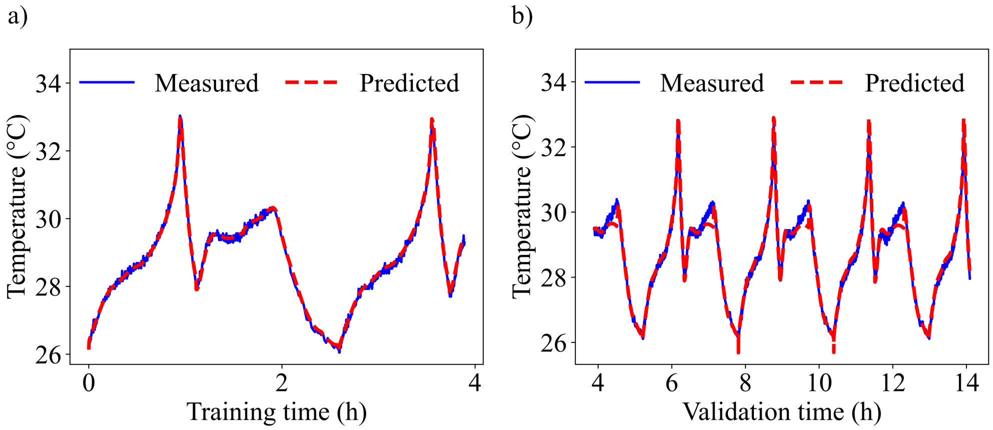

Figure S6: NMC cell around room temperature prediction: (a) training, and (b) validation. The initial cell temperature was 25 °C.

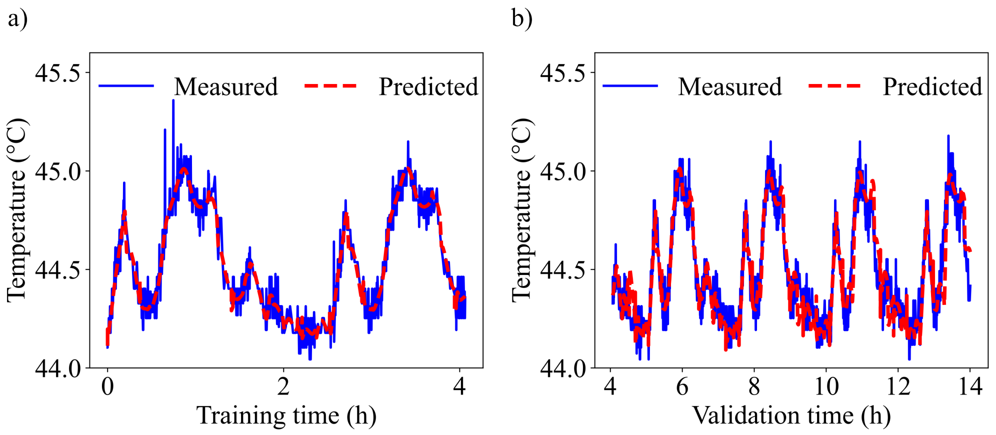

Figure S7: LFP cell high temperature prediction: (a) training, and (b) validation. The initial cell temperature was 45 °C.

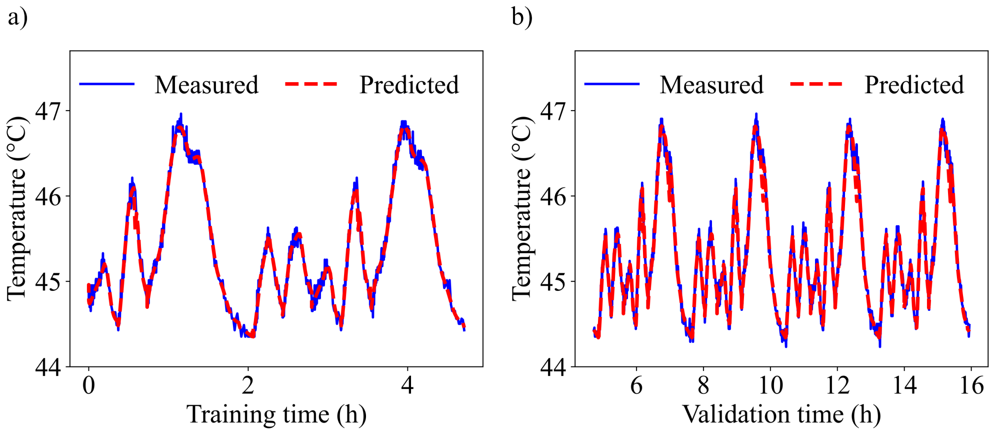

Figure S8: LCO cell high temperature prediction: (a) training, and (b) validation. The initial cell temperature was 45 °C.

a) b)

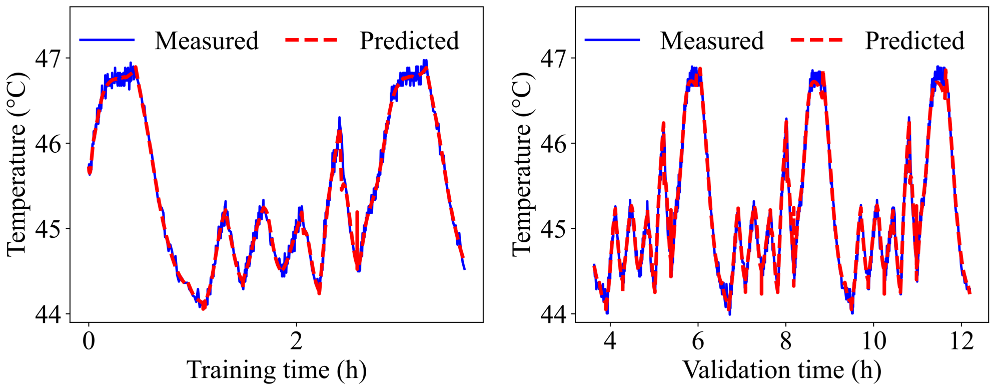

Figure S9: NMC cell high temperature prediction: (a) training, and (b) validation. The initial cell temperature was 45 °C.

3 Temperature predictions using 50% of the dataset

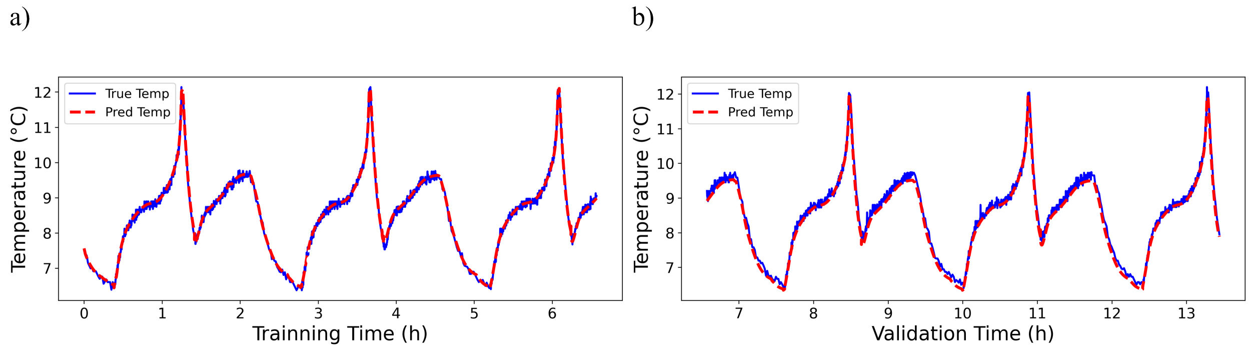

Figure S10: Testing of LCO cell: **(a)** training, and **(b)** validation. The initial cell temperature was 5 °C.

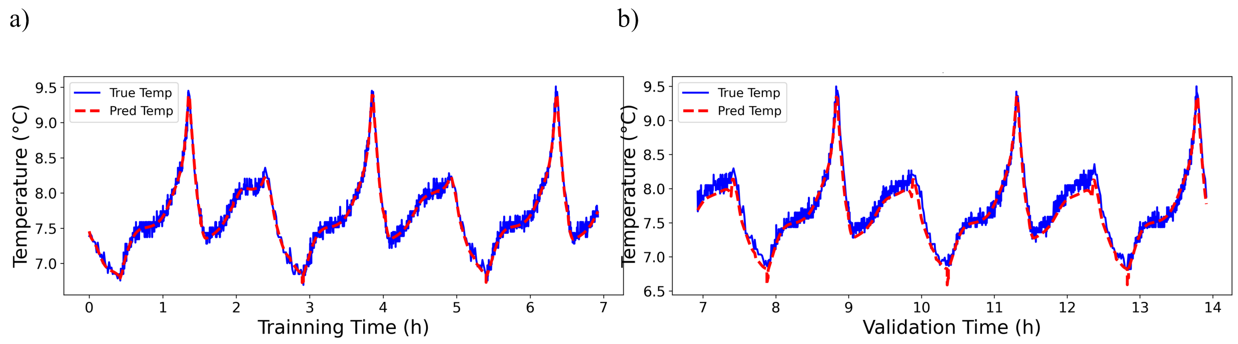

Figure S11: Testing of LFP cell: **(a)** training, and **(b)** validation. The initial cell temperature was 5 °C.

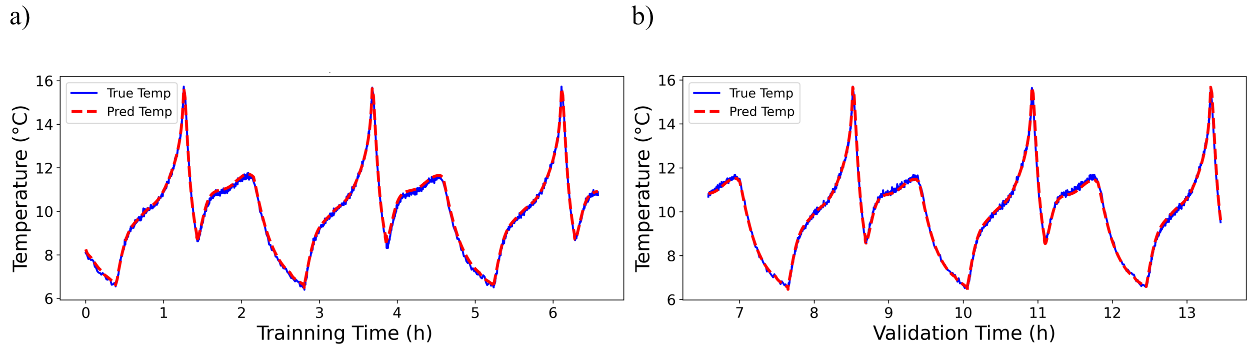

Figure S12: Testing of NMC cell: **(a)** training, and **(b)** validation. The initial cell temperature was 5 °C.

a) b)

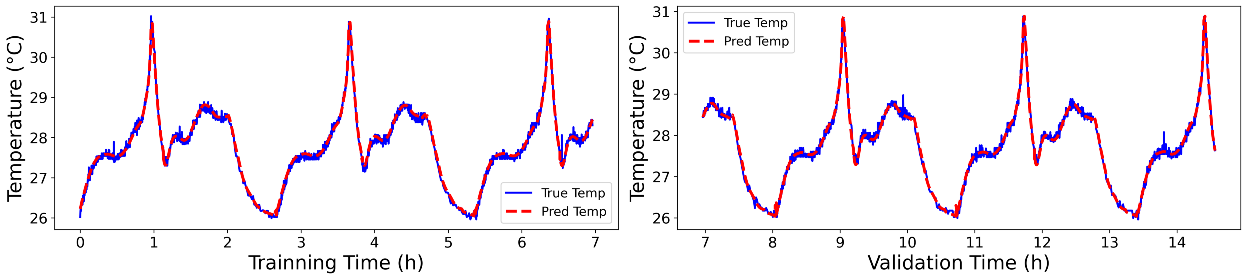

Figure S13: Testing of LCO cell: **(a)** training, and **(b)** validation. The initial cell temperature was 25 °C.

a) b)

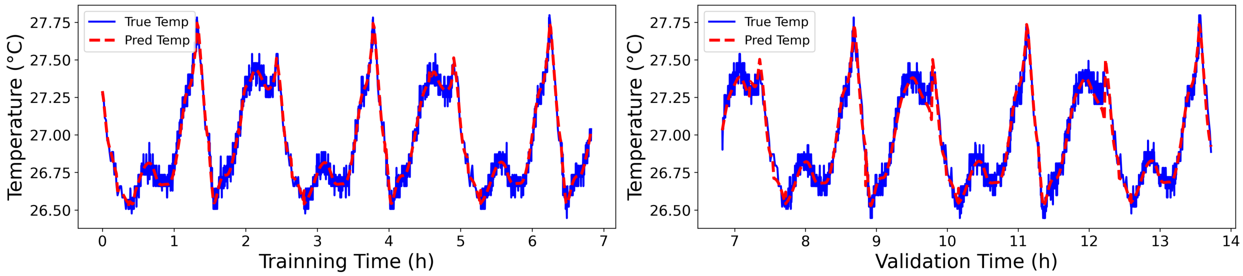

Figure S14: Testing of LFP cell: **(a)** training, and **(b)** validation. The initial cell temperature was 25 °C

a) b)

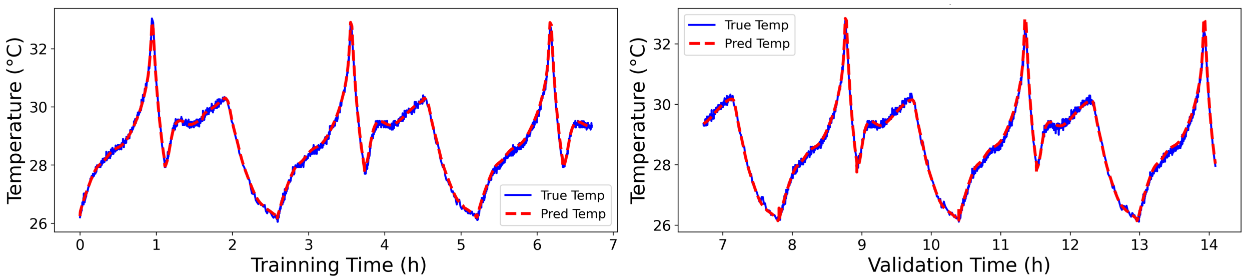

Figure S15: Testing of NMC cell: **(a)** training, and **(b)** validation. The initial cell temperature was 25 °C

a) b)

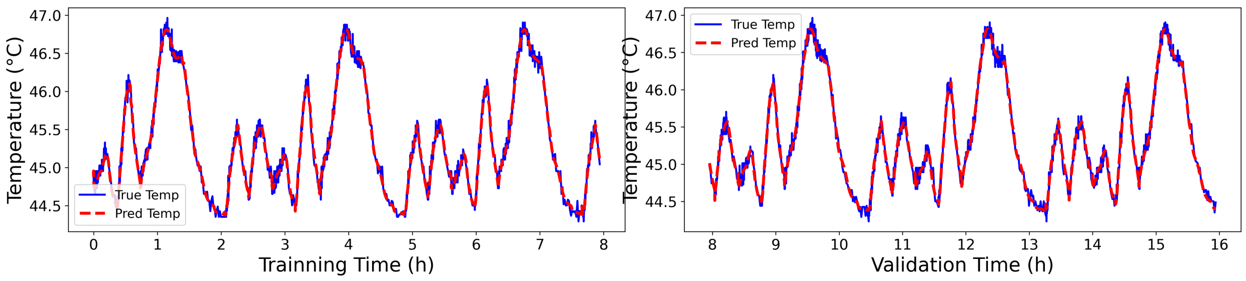

Figure S16: Testing of LCO cell: **(a)** training, and **(b)** validation. The initial cell temperature was 45 °C

a) b)

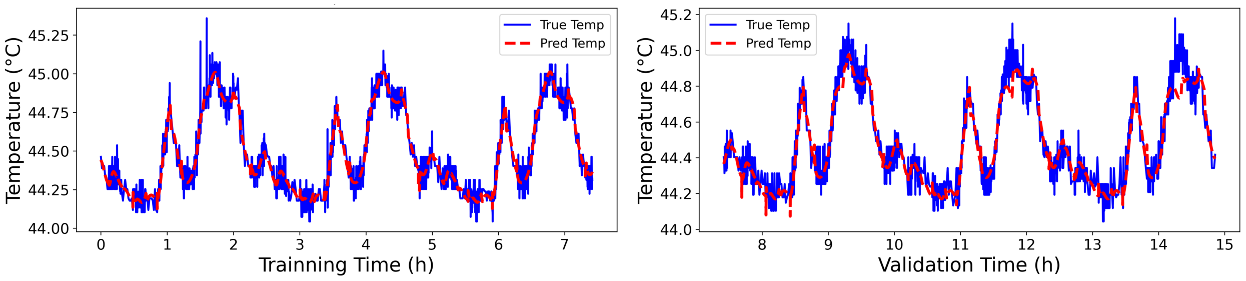

Figure S17: Testing of LFP cell: **(a)** training, and **(b)** validation. The initial cell temperature was 45 °C

a) b)

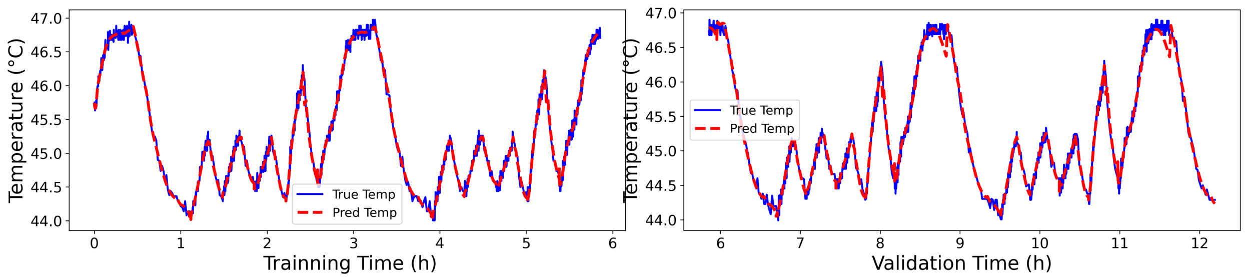

Figure S18: Testing of NMC cell: **(a)** training, and **(b)** validation. The initial cell temperature was 45 °C

3.0.1 PINN vs FCN

To assess the performance of the proposed PINN approach, a comparison was carried out against a standard fully connected network (FCN) using a limited training dataset and errors are presented in table S1. The comparison was conducted across three different chemistries and temperatures. The training size of 30% used for the training, and the remaining portion of the selected dataset size was used for validation. The PINN and the FCN baseline share the exact same dataset tanning/ validation splits.

Table S1: Baseline Method performance for different battery chemistries and temperatures using 30% of the dataset for training. Errors are in °C.

**Chemistry**	**Temp. (°C)**	**Train MAE**	**Train RMSE**	**Val MAE**	**Val RMSE**
NMC	5	0.35	0.58	9.99	11.34
NMC	25	0.23	0.40	16.28	18.37
NMC	45	0.20	0.32	20.95	24.30
LFP	5	0.09	0.18	2.16	2.64
LFP	25	0.13	0.18	5.99	7.12
LFP	45	0.16	0.21	21.08	24.44
LCO	5	0.17	0.35	6.69	7.56
LCO	25	0.19	0.32	15.57	17.72
LCO	45	0.36	0.55	7.02	9.04

4 Sensitivity analysis, cross-validation and ablation study

Table S2: Sensitivity analysis of LCO temperature prediction to effective surface area using 30% training data.

Temperature (°C)	Surface area (m^2)	Train MAE (°C)	Train RMSE (°C)	Val MAE (°C)	Val RMSE (°C)
5	0.0025	0.11	0.13	0.07	0.09
5	0.0042	0.11	0.13	0.07	0.09
25	0.0025	0.07	0.08	0.07	0.10
25	0.0042	0.06	0.08	0.09	0.14
45	0.0025	0.05	0.07	0.07	0.10
45	0.0042	0.05	0.07	0.11	0.23

Table S3: Cross-validation study for LCO cells trained on one chemistry and validated on unseen chemistries.

Temperature (°C)	Trained on Chemistry	Validated on Chemistry	Val MAE (°C)	Val RMSE (°C)
45	LCO	NMC	0.29	0.36
45	LCO	LFP	1.49	1.57
25	LCO	NMC	1.24	1.38
25	LCO	LFP	0.94	1.09
5	LCO	NMC	1.79	2.02
5	LCO	LFP	1.55	1.72

Table S4: Local error for LCO cells (30% training data).

Temperature (°C)	Chemistry	Proposed equation Max local error (°C)	Without radiative term Max local error (°C)	Without entropy term Max local error (°C)
5	LCO	0.74	0.42	0.66
45	LCO	0.64	1.13	1.30

5 Conceptual comparison

Table S5 summarizes the main differences between physics-based models, data-driven approaches, and physics-informed neural networks [2,3]. The comparison focuses on the use of physical laws, data needs, parameter identification, and sensitivity to noisy measurements. This overview helps position the proposed method within existing modeling frameworks and clarifies the practical trade-offs among these approaches.

Table S5: Conceptual comparison between physics-based models, data-driven models, and physics-informed neural networks(PINNs)[2,3]

**Characteristic**	**Physics-Based**	**Data-Driven**	**PINN**
Use of physical laws	Fully governed by physical equations	No explicit physics included	Physical constraints embedded in learning
Data requirement	Limited experimental data	Large labeled datasets	Moderate data
Parameter identification	Explicit parameter estimation required	Not applicable	Partial parameter identification acceptable
Sensitivity to noisy or incomplete data	Can degrade model calibration	Often sensitive to noise	Improved robustness through physics constraints

References

[1] H. M. Barkholtz, A. Fresquez, B. R. Chalamala, and S. R. Ferreira, "A database for comparative electrochemical performance of commercial 18650-format lithium-ion cells," *Journal of The Electrochemical Society*, vol. 164, no. 12, p. A2697, 2017.

[2] G. Cho, M. Wang, Y. Kim, J. Kwon, and W. Su, "A physics-informed machine learning approach for estimating lithium-ion battery temperature," *IEEE Access*, vol. 10, pp. 88 117–88 126, 2022.

[3] G. E. Karniadakis, I. G. Kevrekidis, L. Lu, P. Perdikaris, S. Wang, and L. Yang, "Physics-informed machine learning," *Nature Reviews Physics*, vol. 3, no. 6, pp. 422–440, 2021.
